# Efficacy of immunotherapy and targeted therapy for unresectable hepatocellular carcinoma: analysis of survival curves

**DOI:** 10.3389/fmed.2026.1774216

**Published:** 2026-04-23

**Authors:** Wan Zhou, Pan Xu

**Affiliations:** 1 Department of Gastroenterology, University-Town Hospital of Chongqing Medical University, Chongqing, China; 2 Department of Clinical Laboratory, Medical Sciences Research Center, University-Town Hospital of Chongqing Medical University, Chongqing, China

**Keywords:** immune checkpoint inhibitors, immunotherapy, parametric survival analysis, targeted therapy, tyrosine kinase inhibitors, unresectable hepatocellular carcinoma

## Abstract

**Background and objectives:**

Hepatocellular carcinoma is the third leading cause of cancer-related death worldwide. Because many patients present with unresectable disease at diagnosis and are therefore not candidates for curative resection, systemic therapy has become the cornerstone of first-line treatment for unresectable hepatocellular carcinoma (uHCC). We used parametric survival models to assess the efficacy of immunotherapy and targeted therapy as first-line strategies for treating uHCC, based on an analysis of the evidence from phase III trials, including phase II–III trials from which randomized phase III cohort data could be extracted.

**Methods:**

A systematic literature search of PubMed, Embase, and the Cochrane Library was conducted to identify randomized controlled trials reporting overall survival (OS) and progression-free survival (PFS) as Kaplan–Meier curves (KM curves). Individual patient data (IPD) were reconstructed from the published curves using digitization methods, and a pooled analysis of parametric survival curves was performed within a Bayesian framework to estimate time-dependent hazard ratios (HRs) and survival probabilities.

**Results:**

Fifteen randomized trials involving patients with uHCC were included in the main analysis, and an additional sensitivity analysis explored the potential influence of CheckMate-9DW. The log-normal model provided the best fit for both OS and PFS data. Time-dependent HR analyses indicated clear departures from the proportional hazards (PH) assumption, suggesting that treatment effects varied over time. Combination regimens generally demonstrated more favorable survival outcomes than monotherapies. Among all evaluated treatments, finotonlimab plus bevacizumab biosimilar consistently showed the highest predicted OS and PFS probabilities across multiple time points.

**Conclusion:**

Combination regimens generally showed more favorable survival outcomes than monotherapies in first-line uHCC. Parametric survival modeling suggested that treatment effects varied over time and provided additional insights beyond conventional approaches based on constant HRs. Among the evaluated regimens, finotonlimab plus bevacizumab biosimilar showed a highly favorable efficacy profile; however, long-term comparisons should be interpreted cautiously because they depend partly on extrapolated estimates.

## 1 Introduction

Hepatocellular carcinoma (HCC) is one of the most common causes of cancer-related mortality (1). Surgical resection remains the primary curative treatment for HCC. However, many patients are diagnosed at an advanced stage, often with macrovascular invasion, extrahepatic spread, multifocal disease, or impaired underlying liver function, and are therefore not suitable candidates for curative resection (2). Immunotherapy and targeted therapy have emerged as promising and effective therapeutic strategies for uHCC. In this setting, immunotherapy mainly refers to immune checkpoint inhibitors (ICIs) targeting the programmed cell death protein 1/programmed cell death ligand 1 (PD-1/PD-L1) or cytotoxic T-lymphocyte-associated antigen 4 (CTLA-4) pathways, whereas targeted therapy primarily includes anti-angiogenic agents and tyrosine kinase inhibitors (TKIs). These agents are used either alone or in combination as first-line systemic treatment for uHCC (3).

As multiple first-line systemic options have become available, comparative evaluation of their relative efficacy has become increasingly important. Network meta-analysis (NMA) is a useful tool in this setting because it allows simultaneous comparison of multiple treatments across a connected evidence network, even when head-to-head trials are limited. Two NMAs have evaluated first-line interventions for uHCC using differences in overall survival (OS) and progression-free survival (PFS) as measures of relative efficacy (4, 5). Previous NMAs have used hazard ratios (HRs) as the effect size, indicating that combinations of PD-1/PD-L1 inhibitors with multikinase inhibitors, vascular endothelial growth factor (VEGF) inhibitors, or CTLA-4 inhibitors, such as sintilimab plus a bevacizumab biosimilar, atezolizumab plus bevacizumab, or camrelizumab plus rivoceranib, are among the most effective first-line treatment options (6).

Typically, evidence for time-to-event data is summarized using HRs (7). NMAs based on HRs assume that the proportional hazards (PH) assumption holds for each pair of comparators; that is, the HRs between treatment arms remain constant over time. However, this assumption is often violated (8), particularly when the survival curves of different interventions cross, resulting in biased estimates of expected survival. Moreover, extrapolation beyond the trial period is necessary to avoid underestimating long-term survival (9). Parametric survival analysis has been proposed to address these limitations (10). This approach enables more accurate estimation of treatment effects from time-to-event data and allows the modelling of time-varying HRs. By capturing changes in treatment effects over time, the parametric modelling framework can provide additional insights into the temporal patterns of treatment benefit beyond those obtained from conventional NMAs that assume constant HRs. Therefore, this pooled analysis of parametric survival curves was conducted to evaluate the efficacy of immunotherapy and targeted therapy in patients with uHCC.

## 2 Methods

### 2.1 Search strategy

**Figure 1:**
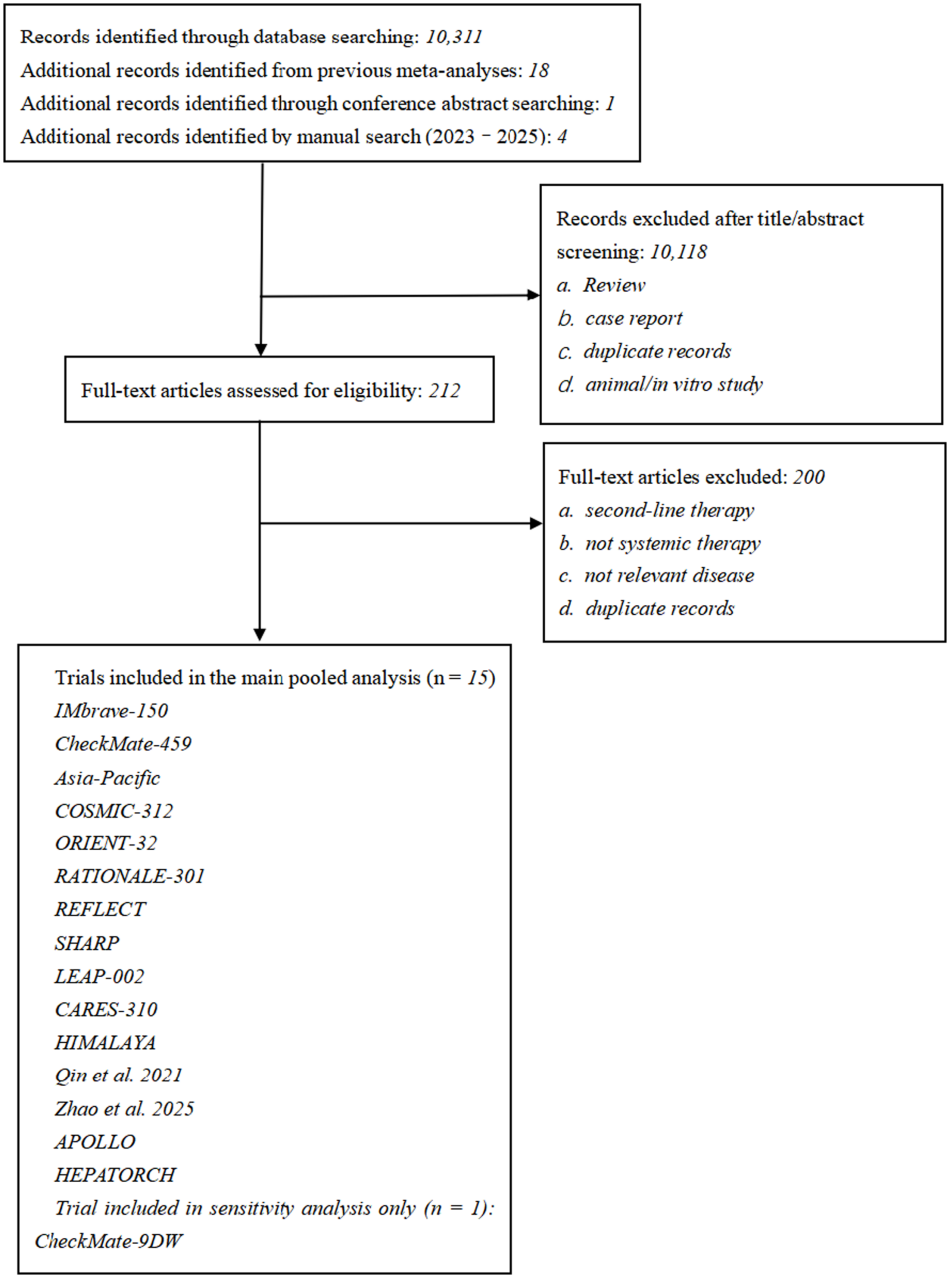
Flow diagram of study selection.

The systematic retrieval of eligible studies was conducted from January 1, 2008 to March 1, 2026. The PubMed, Embase, and Cochrane Library databases were searched using the search terms “advanced hepatocellular carcinoma,” “unresectable hepatocellular carcinoma,” and “trial, study, randomized clinical trial, or randomized controlled trial.” Conference abstracts from the American Society of Clinical Oncology and European Society of Medical Oncology were also reviewed. The detailed search strategy for each database is provided in Supplementary Appendix S1. The numbers of records identified from each source and the subsequent study selection process are summarized in Figure 1.

### 2.2 Selection criteria

Studies were considered eligible if they met all of the following criteria: (1) phase III randomized controlled trials, or phase II-III trials from which data from the randomized phase III cohort could be extracted; (2) adult patients with unresectable, advanced, or metastatic hepatocellular carcinoma who had not received previous systemic therapy; (3) evaluation of first-line systemic treatment with immunotherapy and/or targeted therapy; (4) reporting OS and/or PFS as KM curves, allowing reconstruction of individual patient data; and (5) availability of sufficient published data for extraction. Studies investigating second-line or later-line therapy, locoregional therapy alone, non-systemic interventions, non-randomized designs, duplicate reports, or studies without extractable survival curves were excluded. The present analysis focused specifically on first-line systemic treatment for unresectable, advanced, or metastatic HCC and was not intended to address treatment strategies for resectable or early-stage disease.

### 2.3 Data extraction and quality assessment

WZ and PX independently performed data extraction and quality assessment. For each study that met the selection criteria, information on study design, patient characteristics, and treatment regimens was extracted. The primary data extracted for modelling were digitized KM curves for OS and PFS for each treatment arm, from which individual patient data (IPD) were reconstructed. Reported HRs and corresponding confidence intervals (CIs) for OS and PFS were also extracted as descriptive trial-level summary measures. WebPlotDigitizer^1^ was used to extract data points from the KM curves. Risk of bias was assessed according to the Cochrane Handbook (version 5.1.0) (11).

### 2.4 Statistical analysis

Guyot’s iterative algorithm was applied to digitized published KM curves to reconstruct IPD (12), which were then used for the survival analyses. The survival analyses were performed using the R packages survHE (version 2.0.52) and survHEhmc (version 0.0.11) in R software (version 4.3.2; R Core Team, Vienna, Austria). Parametric survival models were fitted within a Bayesian framework using a fixed-effect structure, with flat priors assigned to all model parameters. The following standard parametric survival functions were compared: exponential, Weibull, Gamma, log-logistic, and log-normal distributions. Model fitting was conducted using Hamiltonian Monte Carlo (HMC) with three independent chains. For each chain, 4,000 iterations were run with a burn-in period of 2000 iterations.

The deviance information criterion (DIC) was reported as a statistical measure of model fit, and the model with the lowest DIC value was used in subsequent analyses (13). Posterior summaries of the scale and shape parameters were based on the post-warm-up samples. R was also used to calculate HRs, hazard rates [*h(t)*], and survival probabilities over time (14). The ggplot2 package in R was used to visualize the results.

## 3 Results

### 3.1 Characteristics of included clinical trials

**Figure 2:**
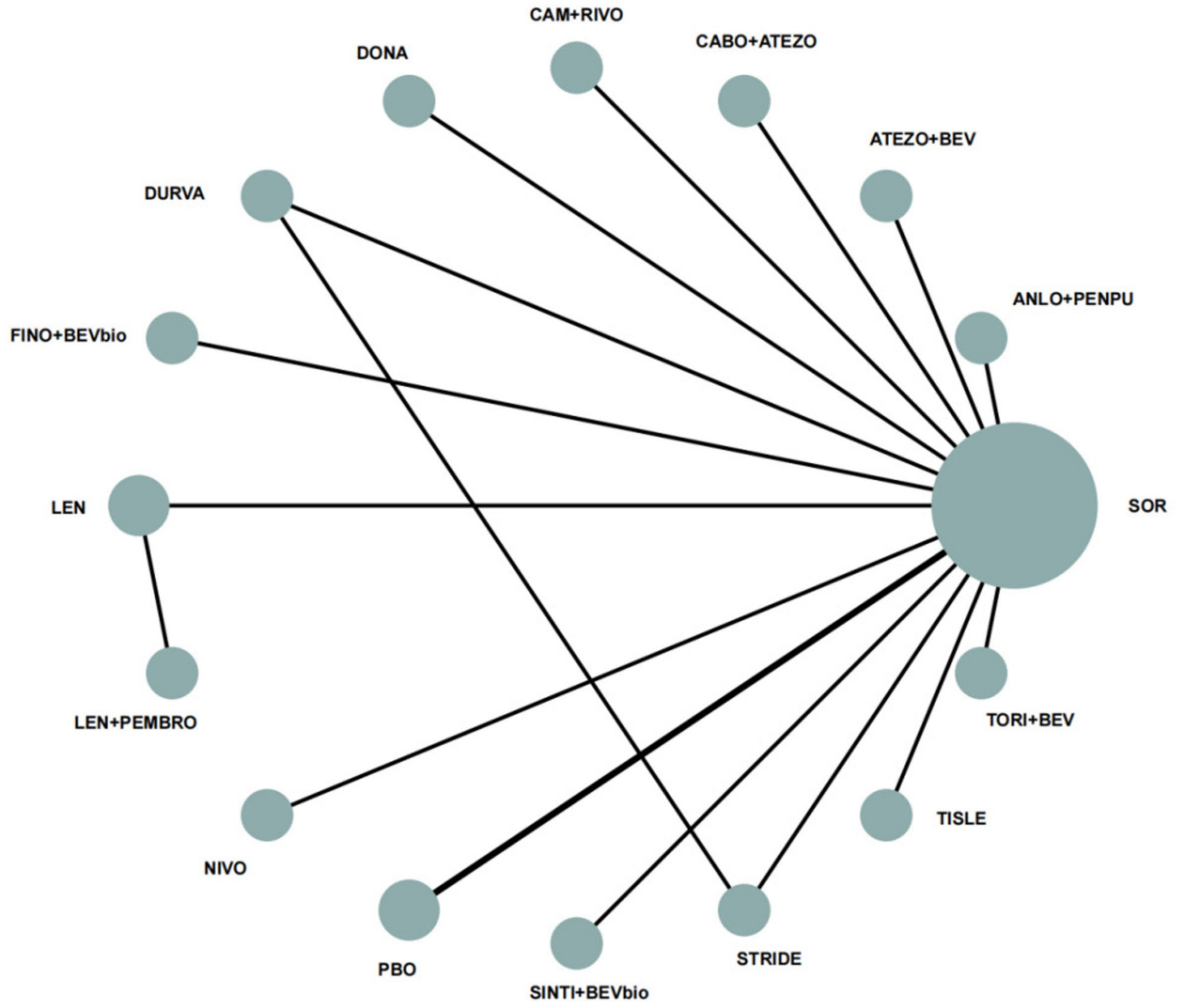
Network plot for OS. OS, overall survival; FINO+BEVbio, finotonlimab + bevacizumab biosimilar; CAM + RIVO, camrelizumab + rivoceranib; TORI+BEV, toripalimab + bevacizumab; LEN + PEMBRO, lenvatinib + pembrolizumab; ATEZO+BEV, atezolizumab + bevacizumab; SINTI+BEVbio, sintilimab + bevacizumab biosimilar; TISLE, tislelizumab; ANLO+PENPU, anlotinib + penpulimab; STRIDE, single tremelimumab regular interval durvalumab regimen; NIVO, nivolumab; LEN, lenvatinib; CABO+ATEZO, cabozantinib + atezolizumab; DURVA, durvalumab; DONA, donafenib; SOR, sorafenib; PBO, placebo.

A total of 10,334 records were identified, of which 10,118 were excluded after title and abstract screening. Of the remaining 212 records assessed in full text, 200 were excluded for prespecified reasons. Fifteen randomized trials were included in the main analysis (15–29), and a subsequent sensitivity analysis explored the potential impact of the CheckMate-9DW trial (30). Figure 1 shows the study selection process. Figure 2 and Supplementary Appendix S2 present the treatment networks for the main OS and PFS analyses, respectively, and Supplementary Appendix S3 summarizes the risk-of-bias assessment. Overall, the included studies showed a low risk of bias in most domains, although performance bias was commonly rated as high in open-label trials.

The characteristics of the included studies are summarized in Table 1. Most trials were phase III randomized controlled trials conducted across multiple regions. Two trials were designed as phase II–III studies (20, 26); however, only data from the phase III randomized cohorts were included in the present analysis. A substantial proportion of participants were from Asia, as several trials were conducted predominantly in Asian populations.

**Table 1:** Characteristics of the trials considered in the present study.

Study	Treatment	Patients, n	Asia, %	HBV, %	HCV, %	Child (A), %	BCLC-C, %	Maximum Follow-up	OS		PFS	
									Median OS	Constant HR (CI)	Median PFS	Constant HR (CI)
Cheng AL et al. (17) IMbrave-150	Atezolizumab + Bevacizumab	336	39.6%	49%	21%	100%	62%	29 mo	19.2	0.66 (0.52–0.85)	6.9	0.65 (0.53–0.81)
	Sorafenib	165	41.2%	46%	22%	100%	62%		13.4		4.3	
Kudo et al. (19) REFLECT	Lenvatinib	478	70%	53%	19%	99%	64%	40 mo	13.6	0.92 (0.79–1.06)	7.4	0.66 (0.57–0.77)
	Sorafenib	476	68%	48%	26%	99%	63%		12.3		3.7	
Yau et al. (25) CheckMate-459	Nivolumab	371	44%	31%	23%	98%	73%	39 mo	16.4	0.85 (0.72–1.02)	3.7	0.93 (0.79–1.10)
	Sorafenib	372	45%	31%	23%	96%	70%		14.7		3.8	
Cheng et al. (16) Asia-Pacific	Sorafenib	150	100%	71%	11%	97.3%	95%	20 mo	6.5	0.68 (0.5–0.93)	NR	NR
	Placebo	76	100%	78%	3.9%	97.4%	96%		4.2		NR	
Llovet et al. (29) SHARP	Sorafenib	299	0	19%	29%	95%	82%	17 mo	10.7	0.69 (0.55–0.87)	NR	NR
	Placebo	303	0	18%	27%	98%	83%		7.9		NR	
Kelley et al. (18) COSMIC-312	Cabozantinib + Atezolizumab	432	29%	29%	31%	100%	68%	27 mo	15.4	0.90 (96% CI 0.69–1.18)	6.8	0.63 (99% CI 0.44–0.91)
	Sorafenib	217	33%	31%	32%	100%	67%		15.5		4.2	
Llovet JM et al. (28) LEAP-002	Lenvatinib + Pembrolizumab	395	30.6%	48.6%	23.8%	99.5%	78.5%	40 mo	21.2	0.84 (0.708–0.997)	8.2	0.834 (0.712–0.978)
	Lenvatinib	399	30.8%	48.4%	21.8%	99.5%	75.7%		19		8.1	
Qin et al. (21) CARES-310	Camrelizumab + Rivoceranib	272	83%	76%	8%	87%	86%	32 mo	22.1	0.62 (0.49–0.80)	5.6	0.52 (0.41–0.65)
	Sorafenib	271	83%	73%	11%	85%	85%		15.2		3.7	
Abou-Alfa et al. (15) HIMALAYA	Tremelimumab + Durvalumab (STRIDE)	393	39.7%	31%	28%	98.5%	62%	46 mo	16.43	0.78 (96.02% CI 0.65–0.93)	3.78	0.90 (0.77–1.05)
	Durvalumab	389	42.9%	31%	28%	97.7%	61%		16.56	0.86 (95.67% CI 0.73–1.03)	3.65	1.02 (0.88–1.19)
	Sorafenib	389	40.1%	31%	27%	97.4%	62%		13.77	/	4.07	/
Qin et al. (24) RATIONALE-301	Tislelizumab	342	62.9%	59.4%	13.5%	99.4%	79.5%	54 mo	15.9	0.85 (0.712–1.019)	2.1	1.11 (0.92–1.33)
	Sorafenib	332	63.3%	62%	11.7%	100%	75.9%		14.1		3.4	
Ren et al. (22) ORIENT-32	Sintilimab + Bevacizumab biosimilar	380	100%	94%	1.6%	96%	85%	15 mo	NR	0.57 (0.43–0.75)	4.6	0·56 (0.46–0.70)
	Sorafenib	191	100%	94%	4.2%	95%	86%		10.4		2.8	
Qin et al. (20)	Donafenib	328	100%	89%	2%	99%	87%	42 mo	12.1	0.831 (0.699–0.988)	3.7	0.909 (0.763–1.082)
	Sorafenib	331	100%	91%	2%	96%	88%		10.3		3.6	
Shi et al. (23) HEPATORCH	Toripalimab + Bevacizumab	162	97%	91%	4%	100%	80%	38 mo	20.0	0.76 (0.58–0.99)	5.8	0.69 (0.53–0.91)
	Sorafenib	164	96%	89%	5%	100%	77%		14.5		4.0	
Yau et al. (30) CheckMate-9DW	Nivolumab + Ipilimumab	335	40%	34%	27%	100%	73%	48 mo	23.7	0.79 (0.65–0.96)	9.1	0.87 (0.72–1.06)
	Lenvatinib or Sorafenib	333	44%	35%	29%	99.7%	73%		20.6		9.2	
Zhao et al. (26)	Finotonlimab + Bevacizumab biosimilar	230	100%	90.4%	4.3%	70.9%	80%	24 mo	22.1	0.60 (0.44–0.81)	7.1	0.50 (0.38–0.65)
	Sorafenib	116	100%	86.2%	6.0%	75.0%	80.2%		14.2		2.9	
Zhou et al. (27) APOLLO	Anlotinib + Penpulimab	433	100%	84%	4%	92%	82%	40 mo	16.5	0.69 (0.55–0.87)	6.9	0.52 (0.41–0.66)
	Sorafenib	216	100%	84%	3%	93%	81%		13.2		2.8	

BCLC, Barcelona Clinic Liver Cancer; HBV, hepatitis B virus; HCV, hepatitis C virus; NR, not reported; HR, hazard ratio; OS, overall survival; PFS, progression-free survival; mo, months.

The included trials evaluated a broad range of systemic treatment regimens for uHCC, including targeted therapies, ICIs, and combination regimens such as sorafenib, nivolumab, lenvatinib, cabozantinib plus atezolizumab, atezolizumab plus bevacizumab, sintilimab plus bevacizumab biosimilar, donafenib, tislelizumab, lenvatinib plus pembrolizumab, camrelizumab plus rivoceranib, tremelimumab plus durvalumab (STRIDE), durvalumab, toripalimab plus bevacizumab, nivolumab plus ipilimumab, finotonlimab plus bevacizumab biosimilar, and anlotinib plus penpulimab, with sorafenib being the most common comparator.

In the main analysis, 13 studies contributed both OS and PFS data, whereas 2 studies contributed OS only (16, 29); the additional sensitivity analysis including CheckMate-9DW considered both OS and PFS. Study-specific KM curves for OS and PFS are shown in Supplementary Appendix S4. These curves illustrate the survival patterns across the included trials and served as the basis for reconstructing IPD used in the subsequent parametric survival modelling. Across the included studies, the maximum reported follow-up duration was 54 months.

### 3.2 Parametric survival modelling and time-dependent treatment effects

**Figure 3:**
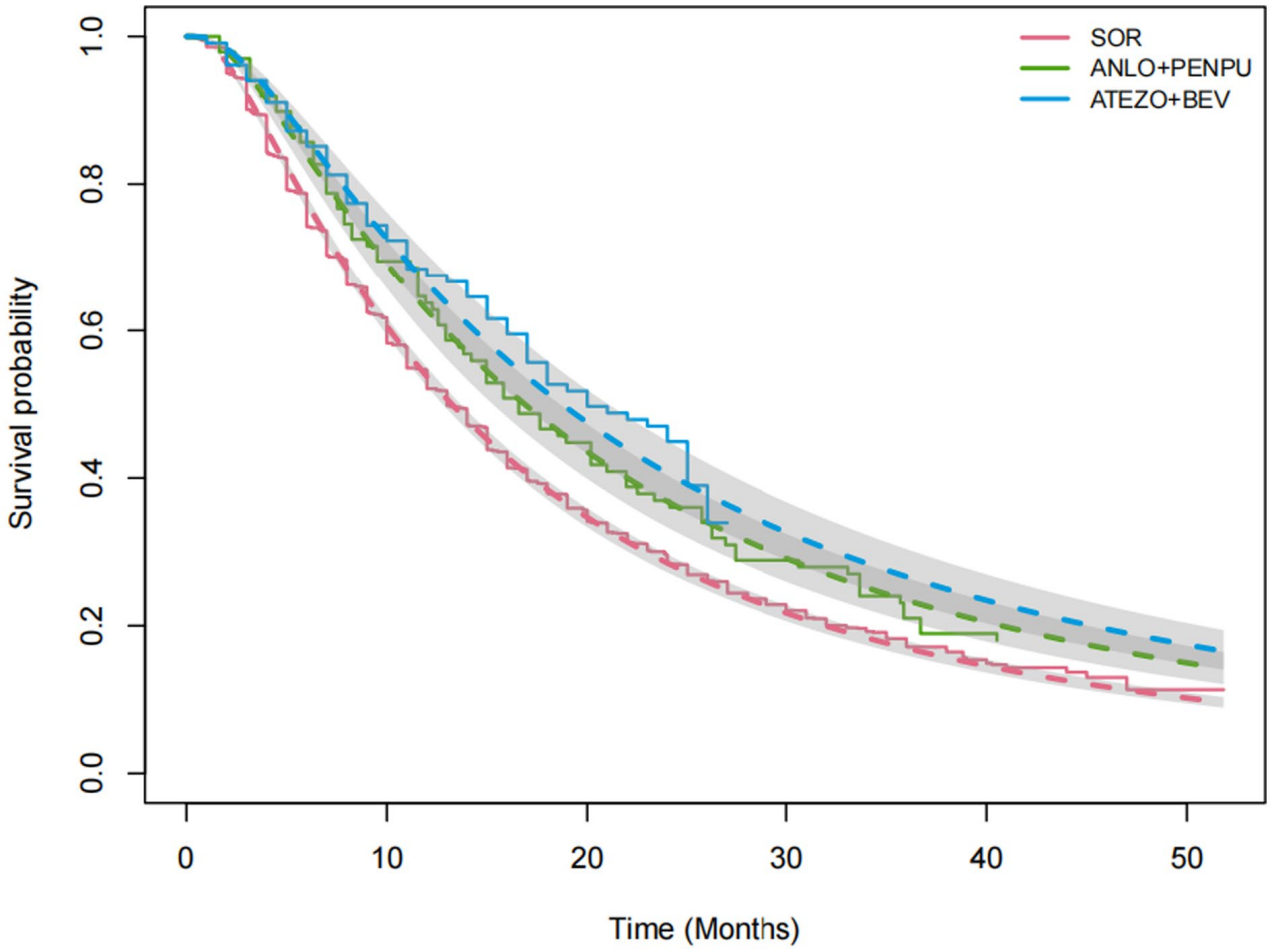
Observed KM curves and corresponding fitted OS curves estimated using the log-normal parametric survival model for selected treatment regimens. OS, overall survival; SOR, sorafenib; ANLO+PENPU, anlotinib + penpulimab; ATEZO+BEV, atezolizumab + bevacizumab.

**Figure 4:**
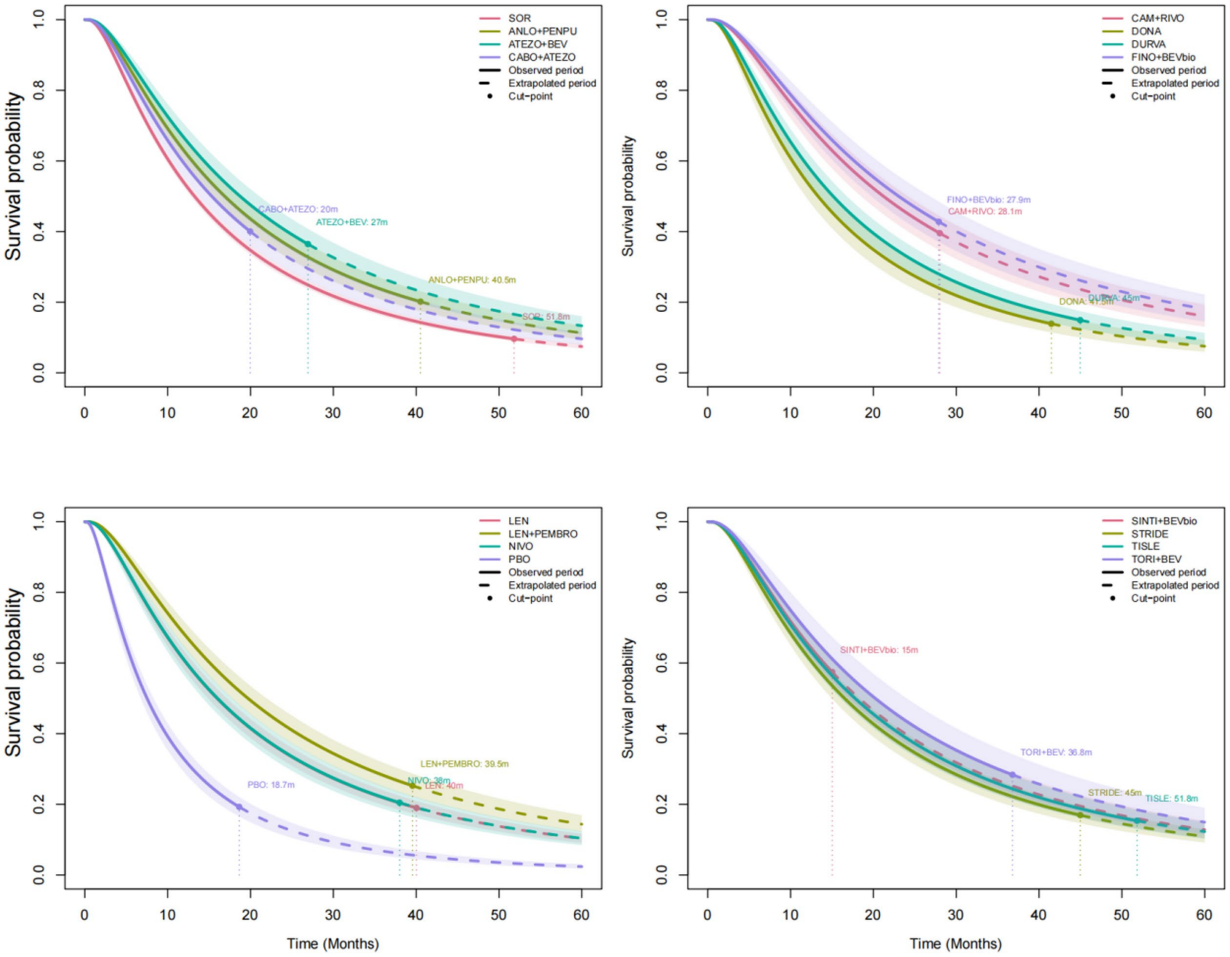
Extrapolated OS curves estimated using the log-normal parametric survival model. OS, overall survival; FINO+BEVbio, finotonlimab + bevacizumab biosimilar; CAM + RIVO, camrelizumab + rivoceranib; TORI+BEV, toripalimab + bevacizumab; LEN + PEMBRO, lenvatinib + pembrolizumab; ATEZO+BEV, atezolizumab + bevacizumab; SINTI+BEVbio, sintilimab + bevacizumab biosimilar; TISLE, tislelizumab; ANLO+PENPU, anlotinib + penpulimab; STRIDE, single tremelimumab regular interval durvalumab regimen; NIVO, nivolumab; LEN, lenvatinib; CABO+ATEZO, cabozantinib + atezolizumab; DURVA, durvalumab; DONA, donafenib; SOR, sorafenib; PBO, placebo.

Sorafenib was used as the reference treatment across the network. For OS, model fit statistics across the five candidate distributions indicated that the log-normal distribution provided the best fit to the data (DIC = 57802.5) (Table 2), followed by the log-logistic, Gamma, Weibull, and exponential distributions. Therefore, subsequent analyses were conducted using the log-normal distribution. The observed KM curves and the corresponding fitted survival curves estimated using the log-normal model for sorafenib, anlotinib plus penpulimab, and atezolizumab plus bevacizumab are shown in Figure 3, demonstrating close agreement between the reconstructed KM data and the parametric estimates (see Supplementary Appendix S5 for the complete set of observed KM curves and fitted survival curves for all treatment regimens). The extrapolated OS curves for all treatment regimens are presented in Figure 4.

**Table 2:** Comparison of the models.

Measure	Outcome	Model 1 Weibull	Model 2 gamma	Model 3 log-logistic	Model 4 log-normal	Model 5 exponential
DIC (fixed-effect)	OS	58312.51	58185.54	57904.21	**57802.5**	58601.33
	PFS	50717.75	50546.8	48827.67	**48694** **.82**	50741.17

DIC, deviance information criterion; OS, overall survival; PFS, progression-free survival. Bold values indicate the lowest DIC value for each outcome and the corresponding best-fitting model.

**Figure 5:**
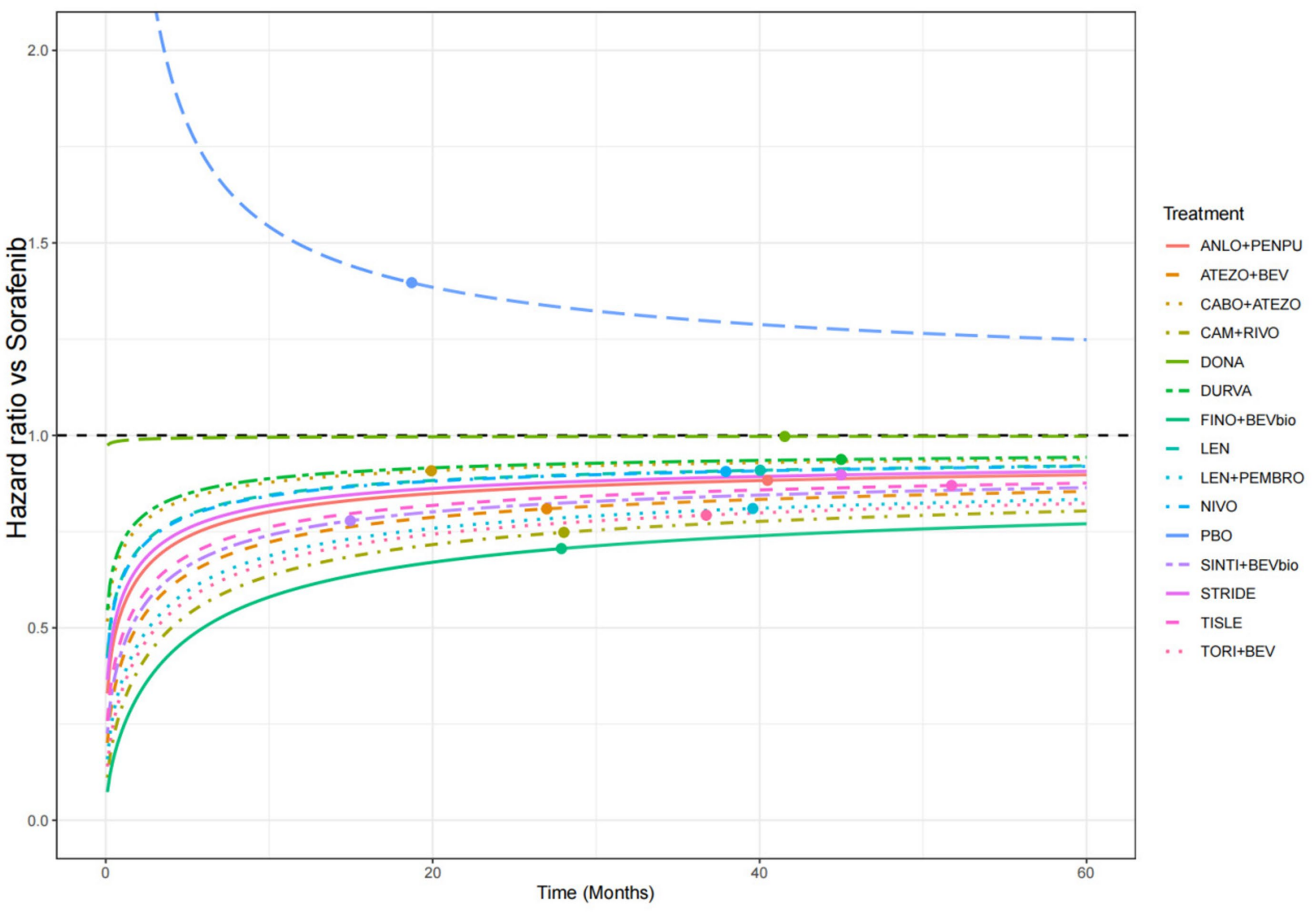
Time-dependent HRs for OS versus sorafenib estimated using the log-normal parametric survival model. HRs, hazard ratios; OS, overall survival; FINO+BEVbio, finotonlimab + bevacizumab biosimilar; CAM + RIVO, camrelizumab + rivoceranib; TORI+BEV, toripalimab + bevacizumab; LEN + PEMBRO, lenvatinib + pembrolizumab; ATEZO+BEV, atezolizumab + bevacizumab; SINTI+BEVbio, sintilimab + bevacizumab biosimilar; TISLE, tislelizumab; ANLO+PENPU, anlotinib + penpulimab; STRIDE, single tremelimumab regular interval durvalumab regimen; NIVO, nivolumab; LEN, lenvatinib; CABO+ATEZO, cabozantinib + atezolizumab; DURVA, durvalumab; DONA, donafenib; SOR, sorafenib; PBO, placebo.

Time-dependent HRs versus sorafenib were further derived from the parametric models to evaluate relative treatment effects over time (Figure 5). The HR curves were extrapolated up to 60 months. Inspection of the time-dependent HR curves indicated clear departures from the PH assumption, suggesting that treatment effects varied over time.

Substantial temporal heterogeneity in hazard trajectories was observed across treatment regimens. Most therapies showed a marked reduction in hazard during the early follow-up period, followed by gradual stabilization over time. Notably, combination regimens tended to demonstrate more favorable hazard profiles than monotherapies throughout follow-up. In particular, the combination of finotonlimab plus bevacizumab biosimilar consistently showed lower HRs than sorafenib across most of the follow-up period.

**Figure 6:**
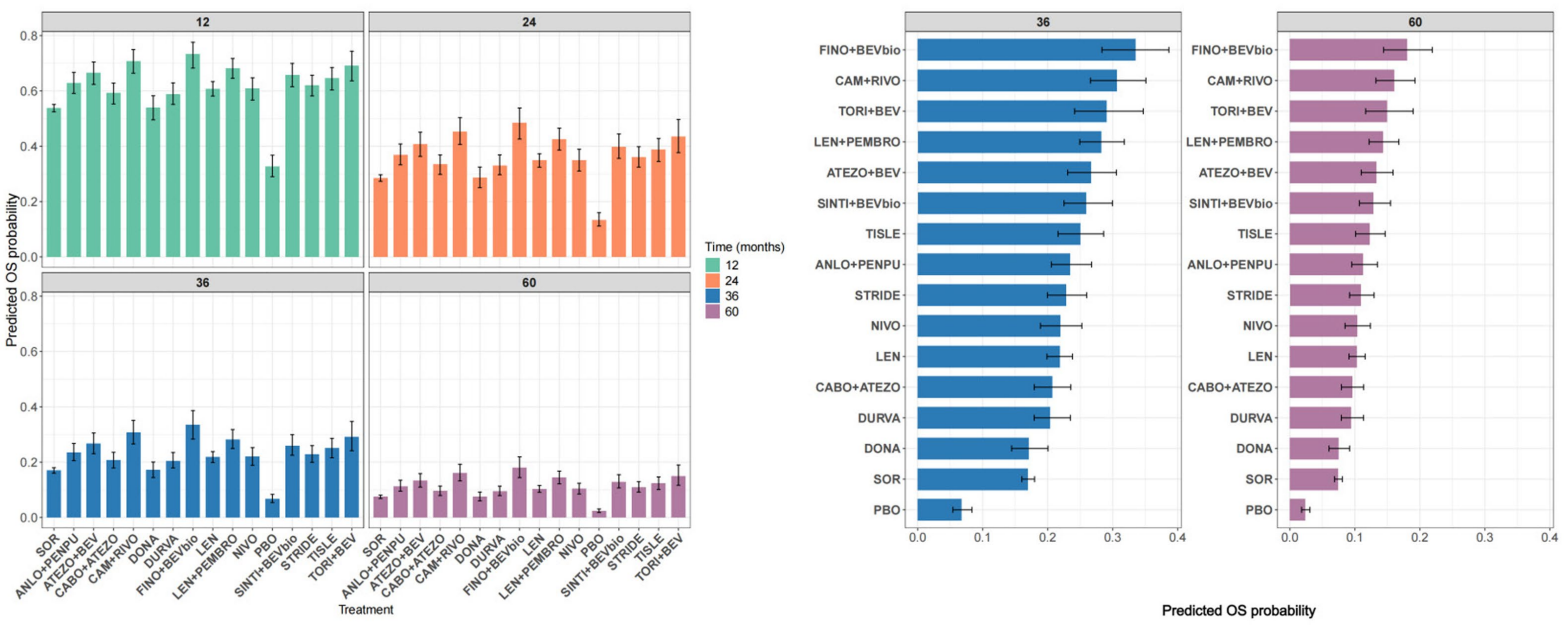
Predicted OS probabilities and ranking of treatments. Left: Predicted OS probabilities at fixed time points. Right: Ranking of treatments according to predicted OS probabilities at 36 and 60 months. OS, overall survival; FINO+BEVbio, finotonlimab + bevacizumab biosimilar; CAM + RIVO, camrelizumab + rivoceranib; TORI+BEV, toripalimab + bevacizumab; LEN + PEMBRO, lenvatinib + pembrolizumab; ATEZO+BEV, atezolizumab + bevacizumab; SINTI+BEVbio, sintilimab + bevacizumab biosimilar; TISLE, tislelizumab; ANLO+PENPU, anlotinib + penpulimab; STRIDE, single tremelimumab regular interval durvalumab regimen; NIVO, nivolumab; LEN, lenvatinib; CABO+ATEZO, cabozantinib + atezolizumab; DURVA, durvalumab; DONA, donafenib; SOR, sorafenib; PBO, placebo.

Predicted OS probabilities at fixed time points (12, 24, 36, and 60 months) were estimated from the parametric models to facilitate comparisons across treatments (Figure 6). These results were broadly consistent with the time-dependent HR patterns described above. Overall, combination therapies tended to demonstrate higher predicted OS probabilities than monotherapies, with the differences becoming more pronounced at later time points.

Among all regimens, finotonlimab plus bevacizumab biosimilar consistently showed the highest predicted survival probabilities and ranked highest across multiple time points. In contrast, cabozantinib plus atezolizumab showed lower predicted survival probabilities than several monotherapies, including tislelizumab, nivolumab, and lenvatinib.

The same modelling framework was applied to the PFS data. Similar to the OS analysis, the log-normal distribution provided the best fit for the PFS data (DIC = 48694.82) (Table 2). The fitted survival curves showed good agreement with the reconstructed KM curves, indicating adequate model performance (Supplementary Appendix S6).

**Figure 7:**
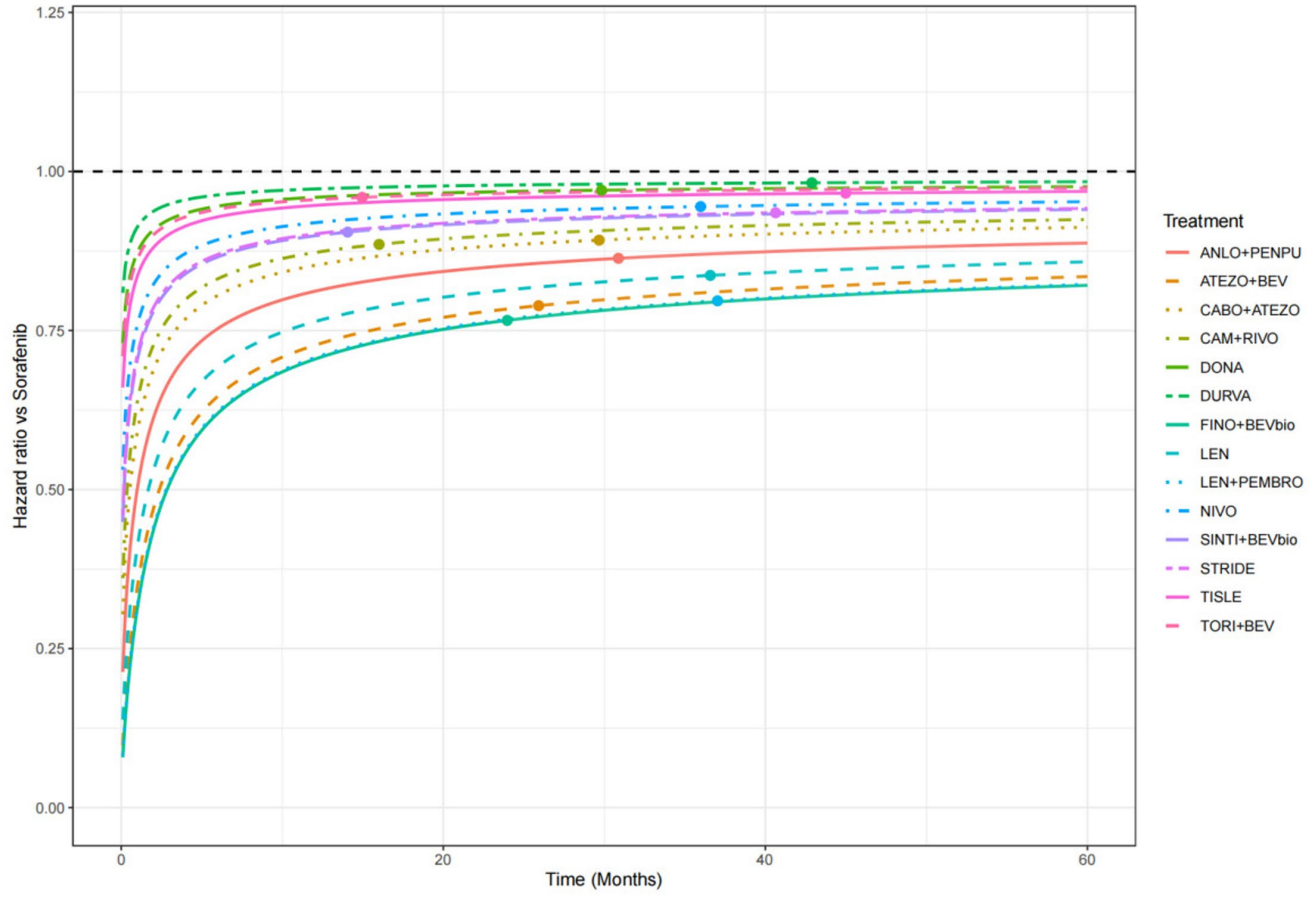
Time-dependent HRs for PFS versus sorafenib estimated using the log-normal parametric survival model. HRs, hazard ratios; PFS, progression-free survival; ANLO+PENPU, anlotinib + penpulimab; ATEZO+BEV, atezolizumab + bevacizumab; CABO+ATEZO, cabozantinib + atezolizumab; CAM + RIVO, camrelizumab + rivoceranib; DONA, donafenib; DURVA, durvalumab; FINO+BEVbio, finotonlimab + bevacizumab biosimilar; LEN, lenvatinib; LEN + PEMBRO, lenvatinib + pembrolizumab; NIVO, nivolumab; SINTI+BEVbio, sintilimab + bevacizumab biosimilar; STRIDE, single tremelimumab regular interval durvalumab regimen; TISLE, tislelizumab; TORI+BEV, toripalimab + bevacizumab.

Time-dependent HRs versus sorafenib derived from the parametric models also suggested that treatment effects varied over time (Figure 7). Overall, combination regimens tended to demonstrate more favorable hazard profiles than monotherapies, consistent with the patterns observed in the OS analysis. Among all evaluated treatments, finotonlimab plus bevacizumab biosimilar consistently showed the most favorable hazard profile relative to sorafenib.

**Figure 8:**
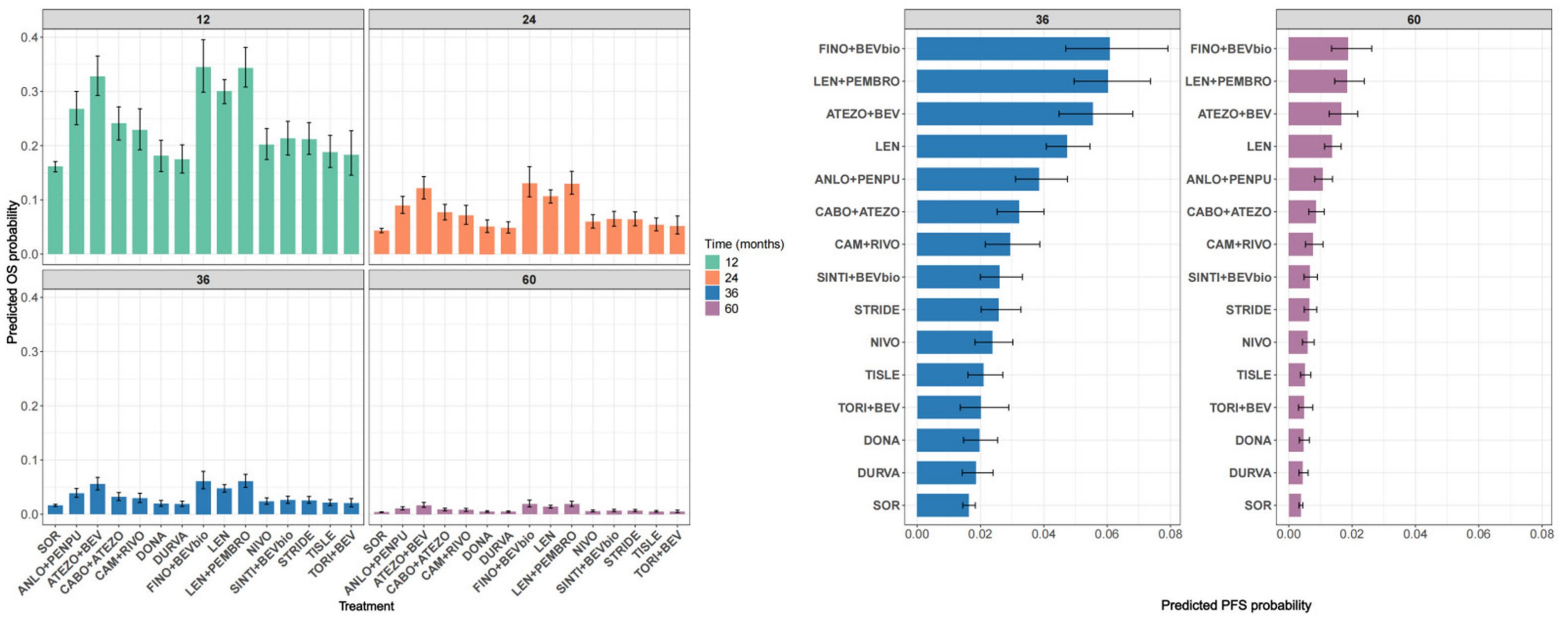
Predicted PFS probabilities and ranking of treatments. Left: Predicted PFS probabilities at fixed time points. Right: Ranking of treatments according to predicted PFS probabilities at 36 and 60 months. PFS, progression-free survival; ANLO+PENPU, anlotinib + penpulimab; ATEZO+BEV, atezolizumab + bevacizumab; CABO+ATEZO, cabozantinib + atezolizumab; CAM + RIVO, camrelizumab + rivoceranib; DONA, donafenib; DURVA, durvalumab; FINO+BEVbio, finotonlimab + bevacizumab biosimilar; LEN, lenvatinib; LEN + PEMBRO, lenvatinib + pembrolizumab; NIVO, nivolumab; SINTI+BEVbio, sintilimab + bevacizumab biosimilar; STRIDE, single tremelimumab regular interval durvalumab regimen; TISLE, tislelizumab; TORI+BEV, toripalimab + bevacizumab.

Predicted PFS probabilities at fixed time points (12, 24, 36, and 60 months) were estimated to facilitate comparisons across treatments (Figure 8). In general, the patterns observed for PFS were broadly consistent with those observed for OS, with combination therapies demonstrating higher predicted survival probabilities than most monotherapies. Finotonlimab plus bevacizumab biosimilar consistently ranked highest across multiple time points. However, some differences in treatment ranking were observed compared with the OS results, particularly at later time points.

### 3.3 Sensitivity analyses

Sensitivity analyses using alternative parametric distributions (log-logistic, gamma, Weibull, and exponential) produced patterns of time-dependent HRs and treatment rankings similar to those of the log-normal model (Supplementary Appendix S7). These findings suggest that the main results were robust to the choice of parametric survival distribution.

An additional sensitivity analysis was conducted to explore the potential impact of including the CheckMate-9DW (2025) trial, in which the control arm consisted of the investigator’s choice of lenvatinib or sorafenib rather than a single comparator. Because this mixed control arm could not be directly incorporated into the treatment network, the trial was excluded from the main analysis. In the sensitivity analysis, the control arm was classified as lenvatinib to enable integration of the study into the network. This approach was considered reasonable because most patients in the control arm of CheckMate-9DW received lenvatinib (approximately 85%), whereas only a minority received sorafenib. Moreover, previous phase III evidence from the REFLECT trial demonstrated that lenvatinib was non-inferior to sorafenib for OS, suggesting that the two agents have broadly comparable efficacy in the first-line setting. The overall patterns of treatment effects and treatment rankings remained largely consistent with those of the main analysis. In particular, finotonlimab plus bevacizumab biosimilar remained the highest-ranked regimen, although nivolumab plus ipilimumab was introduced among the top-ranked regimens (Supplementary Appendices S8–S11).

## 4 Discussion

This study performed a comprehensive comparison of first-line systemic treatment strategies for uHCC based on reconstructed IPD and Bayesian parametric survival models. The results showed that the log-normal distribution provided the best fit for both OS and PFS, indicating that the hazard functions across treatments did not satisfy the proportional hazards assumption. Time-dependent HR analyses further confirmed that the relative treatment effects varied substantially over time.

Overall, combination regimens demonstrated superior efficacy compared with monotherapies, as reflected by more favorable predicted survival probabilities and hazard profiles for both OS and PFS. Among all evaluated treatments, finotonlimab plus bevacizumab biosimilar consistently achieved the highest predicted survival probabilities at multiple time points and maintained the lowest hazard levels throughout most of the follow-up period. These findings suggest that the combination of immunotherapy and anti-angiogenic therapy confers a substantial therapeutic advantage in the first-line treatment of uHCC.

The time-dependent HR curves showed that most treatments exhibited a marked reduction in hazard during the early follow-up period, followed by gradual stabilization. This pattern indicates that conventional approaches based on constant HRs may not adequately capture the true treatment effects. Traditional NMAs typically assume proportional hazards, whereby HRs remain constant over time. However, this assumption is often violated in immunotherapy studies. ICIs are characterized by delayed onset but durable responses, whereas targeted therapies tend to produce earlier but less sustained tumor control. Consequently, the hazard functions of different treatments may evolve over time and even cross. By applying parametric survival modelling, this study was able to directly estimate full survival curves and time-dependent HRs, thereby providing a more accurate characterization of the dynamic treatment effects.

The findings of this study are generally consistent with those reported in several pivotal phase III trials. In recent years, multiple immunotherapy-based combination regimens have demonstrated significant efficacy in the first-line treatment of uHCC. For example, the IMbrave150 trial established that atezolizumab plus bevacizumab significantly improved OS and PFS compared with sorafenib, thereby reshaping the treatment landscape. Subsequent phase III trials have further supported the efficacy of immunotherapy combinations, including sintilimab plus bevacizumab biosimilar, camrelizumab plus rivoceranib, toripalimab plus bevacizumab, and the STRIDE regimen. However, not all combination strategies have yielded consistent benefits. In the COSMIC-312 trial, cabozantinib plus atezolizumab improved PFS but failed to significantly improve OS. Consistent with this finding, the present parametric analysis showed that cabozantinib plus atezolizumab had lower predicted survival probabilities than several other treatments, including tislelizumab, nivolumab, and lenvatinib. In contrast, although the LEAP-002 trial did not meet its predefined statistical significance threshold, lenvatinib plus pembrolizumab still demonstrated clinically meaningful improvements in OS and PFS. In the current analysis, this regimen remained among the top-ranked treatments across multiple time points, suggesting that it may still represent a promising therapeutic option when time-varying treatment effects are considered.

Differences in efficacy among treatment strategies may be explained by their complementary mechanisms of action. ICIs restore antitumor immunity by blocking the PD-1/PD-L1 or CTLA-4 pathways, leading to sustained immune-mediated tumor control (31). Anti-angiogenic therapies, including anti-VEGF antibodies and VEGF receptor (VEGFR)-targeted TKIs, can enhance immunotherapy by normalizing tumor vasculature, reducing the immunosuppressive microenvironment, and promoting immune cell infiltration. Therefore, the combination of ICIs with anti-angiogenic agents is generally considered to have strong biological synergy (32–34). In this study, finotonlimab plus bevacizumab biosimilar consistently demonstrated low hazard levels and favorable long-term survival probabilities (26), supporting this synergistic effect. In addition, differences between PD-1 and PD-L1 inhibitors in immune modulation and target distribution may also contribute to variations in treatment efficacy (35).

Beyond efficacy, the safety profiles of different treatment strategies are also clinically relevant. Although this study primarily focused on survival outcomes, the safety of the major regimens has been well documented in the original randomized trials (see Supplementary Appendix S12). In general, combination therapies are associated with higher rates of adverse events than monotherapies, although substantial heterogeneity exists among different combinations. ICI plus anti-angiogenic antibody regimens tend to have moderate toxicity; for example, in the IMbrave150 trial, the incidence of grade ≥3 treatment-related adverse events was 56% (17), whereas the corresponding rate for finotonlimab plus bevacizumab biosimilar was approximately 52.6% (26). In contrast, ICI plus TKI combinations are typically associated with a higher toxicity burden, as illustrated by the CARES-310 trial, in which the incidence of grade ≥3 adverse events reached 81% (21). ICI monotherapy generally appears to be associated with a lower incidence of severe adverse events than combination regimens, although the reported rates vary across trials. Therefore, in clinical practice, treatment selection should balance efficacy benefits with safety considerations and patient tolerance.

Several limitations of this study should be acknowledged. First, the IPD were reconstructed from digitized KM curves rather than obtained directly from the original trial databases, which may have introduced reconstruction errors. Second, the included studies differed in baseline characteristics, particularly in the proportion of Asian patients, which may have contributed to heterogeneity in treatment effects.

Third, survival outcomes were extrapolated to 60 months, whereas some included studies had relatively short observed follow-up durations. Accordingly, long-term estimates are more uncertain and should be interpreted more cautiously than estimates within the observed follow-up window. Fourth, due to methodological constraints, this study did not perform a formal quantitative analysis of safety outcomes, although existing evidence suggests that the safety profiles of these treatments are generally acceptable. Finally, the number of included studies was limited, and the results should therefore be interpreted with caution.

Despite these limitations, this study systematically integrated the available phase III evidence and applied parametric survival modelling to characterize time-varying treatment effects. The findings further support the important role of immunotherapy combined with anti-angiogenic therapy in the first-line treatment of uHCC. Among all evaluated regimens, finotonlimab plus bevacizumab biosimilar consistently demonstrated the most favorable survival outcomes, suggesting that it may represent a promising therapeutic option. Future studies, including head-to-head trials and real-world evidence, are needed to validate these findings and to identify predictive biomarkers that can guide individualized treatment strategies.

## 5 Conclusion

In conclusion, this study demonstrates that treatment effects for first-line systemic therapies in uHCC are time-dependent and do not conform to the proportional hazards assumption. Parametric survival modelling provides a robust framework for capturing these dynamic treatment effects and offers additional insights beyond conventional approaches based on constant HRs. Combination regimens, particularly immunotherapy combined with anti-angiogenic therapy, consistently showed superior survival outcomes compared with monotherapies. Among the evaluated regimens, finotonlimab plus bevacizumab biosimilar showed a highly favorable efficacy profile; however, long-term rankings should be interpreted cautiously because they depend partly on extrapolated survival estimates and may change as new phase III evidence emerges.

## Data Availability

The original contributions presented in the study are included in the article/Supplementary material, further inquiries can be directed to the corresponding author.

## Footnotes

1
https://apps.automeris.io/wpd4/index.zh_CN.html
