# Supplementary material for: Efficacy of immunotherapy and targeted therapy for unresectable hepatocellular carcinoma: analysis of survival curves
Source: Front Med (Lausanne). 2026 Apr 23;13:1774216. doi: 10.3389/fmed.2026.1774216 (PMC13149074; doi:10.3389/fmed.2026.1774216)
Supplement: Supplementary file 1 [file Supplementary_file_1.docx]

**Contents of Supplementary Appendix**

[Appendix S1. Search strategy 2](#_Toc17318)

[Appendix S2: Network plot for progression-free survival (PFS) 3](#_Toc14798)

[Appendix S3: Risk of bias assessment of included studies 4](#_Toc4620)

[Appendix S4: Reconstructed individual patient data (IPD) 6](#_Toc18594)

[Appendix S5: Observed Kaplan–Meier curves and fitted overall survival (OS) curves estimated using the log-normal parametric survival model 8](#_Toc30813)

[Appendix S6: Observed Kaplan–Meier curves and fitted progression-free survival (PFS) curves estimated using the log-normal parametric survival model 10](#_Toc10546)

[Appendix S7: Fitted survival curves under five parametric distributions 12](#_Toc10489)

[Appendix S8: Sensitivity analysis including CheckMate 9DW: time-varying hazard ratios for overall survival (OS) 13](#_Toc27521)

[Appendix S9: Sensitivity analysis including CheckMate 9DW: overall survival (OS) probabilities and ranking 14](#_Toc13379)

[Appendix S10: Sensitivity analysis including CheckMate 9DW: time-varying hazard ratios for progression-free survival (PFS) 15](#_Toc26399)

[Appendix S11: Sensitivity analysis including CheckMate 9DW: progression-free survival (PFS) probabilities and ranking 16](#_Toc11412)

[Appendix S12: Summary of safety outcomes and major adverse events in patients with uHCC 17](#_Toc29132)

**Appendix S1. Search strategy**

**PubMed**

("hepatocellular carcinoma"[Title/Abstract] OR HCC[Title/Abstract])

AND (unresectable[Title/Abstract] OR advanced[Title/Abstract] OR uHCC[Title/Abstract])

Filters: Clinical Trial; Randomized Controlled Trial

Records identified: 1,491

**Cochrane Central Register of Controlled Trials (CENTRAL)**

("hepatocellular carcinoma":ti,ab,kw OR HCC:ti,ab,kw)

AND (unresectable:ti,ab,kw OR advanced:ti,ab,kw OR uHCC:ti,ab,kw)

Filter: Trials

Records identified: 4,107

**Embase**

('hepatocellular carcinoma':ab,ti OR HCC:ab,ti)

AND (unresectable:ab,ti OR advanced:ab,ti OR uHCC:ab,ti)

AND ([controlled clinical trial]/lim OR [randomized controlled trial]/lim)

Records identified: 4,713

**Additional sources**

Additional records were identified through the following approaches:

* Screening of previous meta-analyses: 18 records

* Conference abstract searches from the European Society for Medical Oncology (ESMO) and the American Society of Clinical Oncology (ASCO): 1 record

* Manual search of relevant literature (2023–2025): 4 records

****Total records identified.**

A total of 10,334 records were identified across all sources prior to screening.

**Appendix S2: Network plot for progression-free survival (PFS)**

**
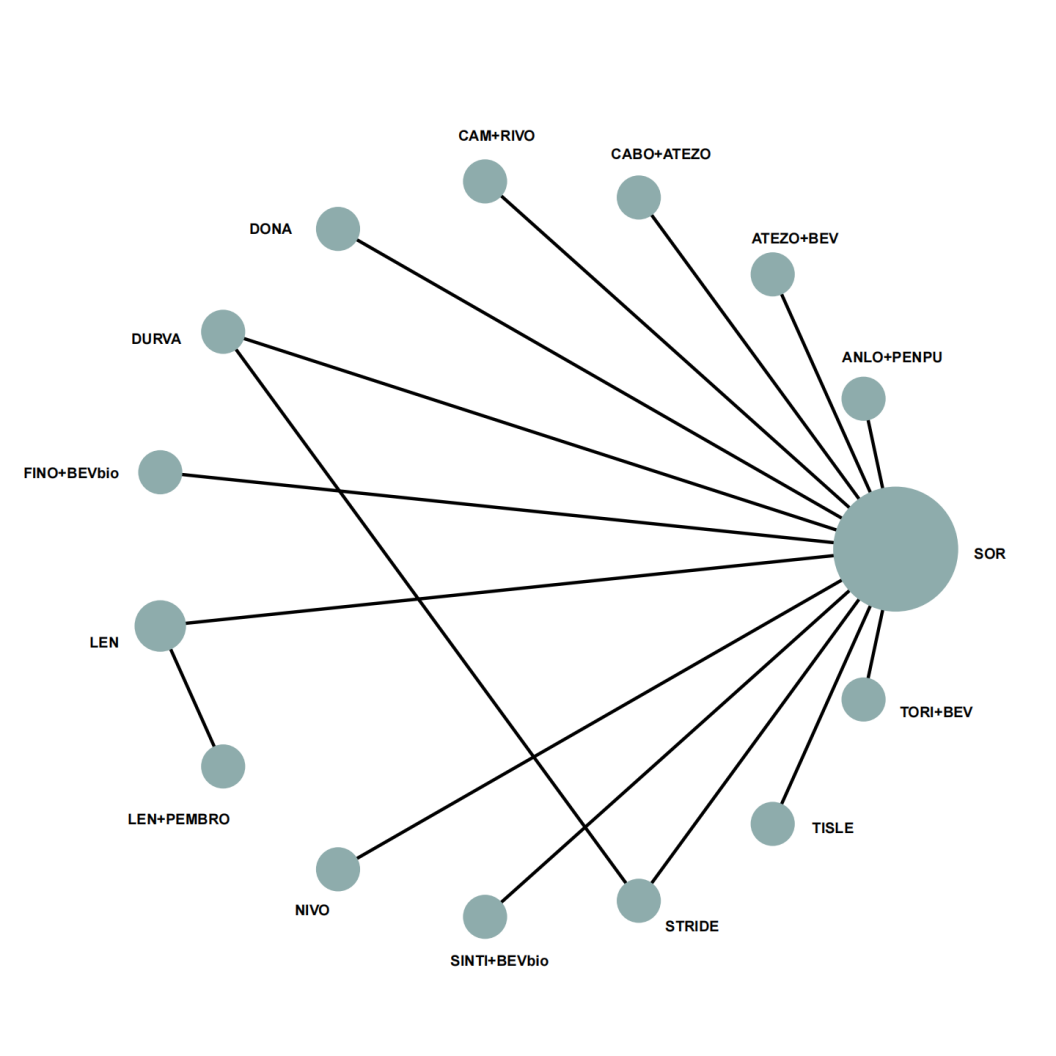
Abbreviations:** FINO+BEVbio = finotonlimab + bevacizumab biosimilar; CAM+RIVO = camrelizumab + rivoceranib; TORI+BEV = toripalimab + bevacizumab; LEN+PEMBRO = lenvatinib + pembrolizumab; ATEZO+BEV = atezolizumab + bevacizumab; SINTI+BEVbio = sintilimab + bevacizumab biosimilar; TISLE = tislelizumab; ANLO+PENPU = anlotinib + penpulimab; STRIDE = single tremelimumab regular interval durvalumab regimen; NIVO = nivolumab; LEN = lenvatinib; CABO+ATEZO = cabozantinib + atezolizumab; DURVA = durvalumab; DONA = donafenib; SOR = sorafenib; PBO = placebo;

**
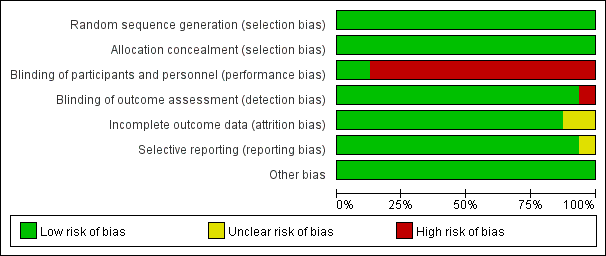
Appendix S3: Risk of bias assessment of included studies**

Appendix S3a: Overall summary of risk of bias


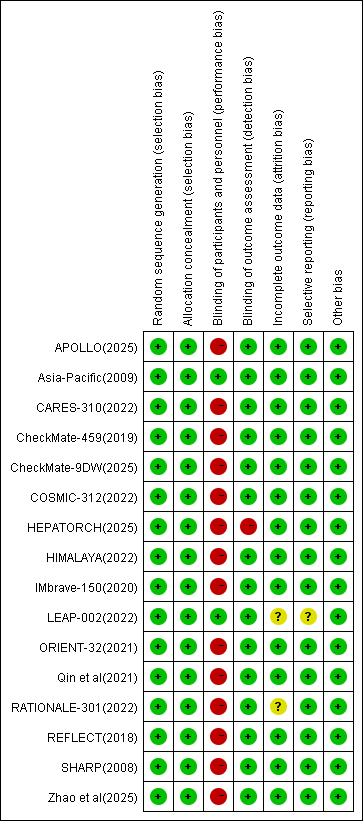


Appendix S3b: Study-level risk of bias assessment

**Appendix S4: Reconstructed individual patient data (IPD)**


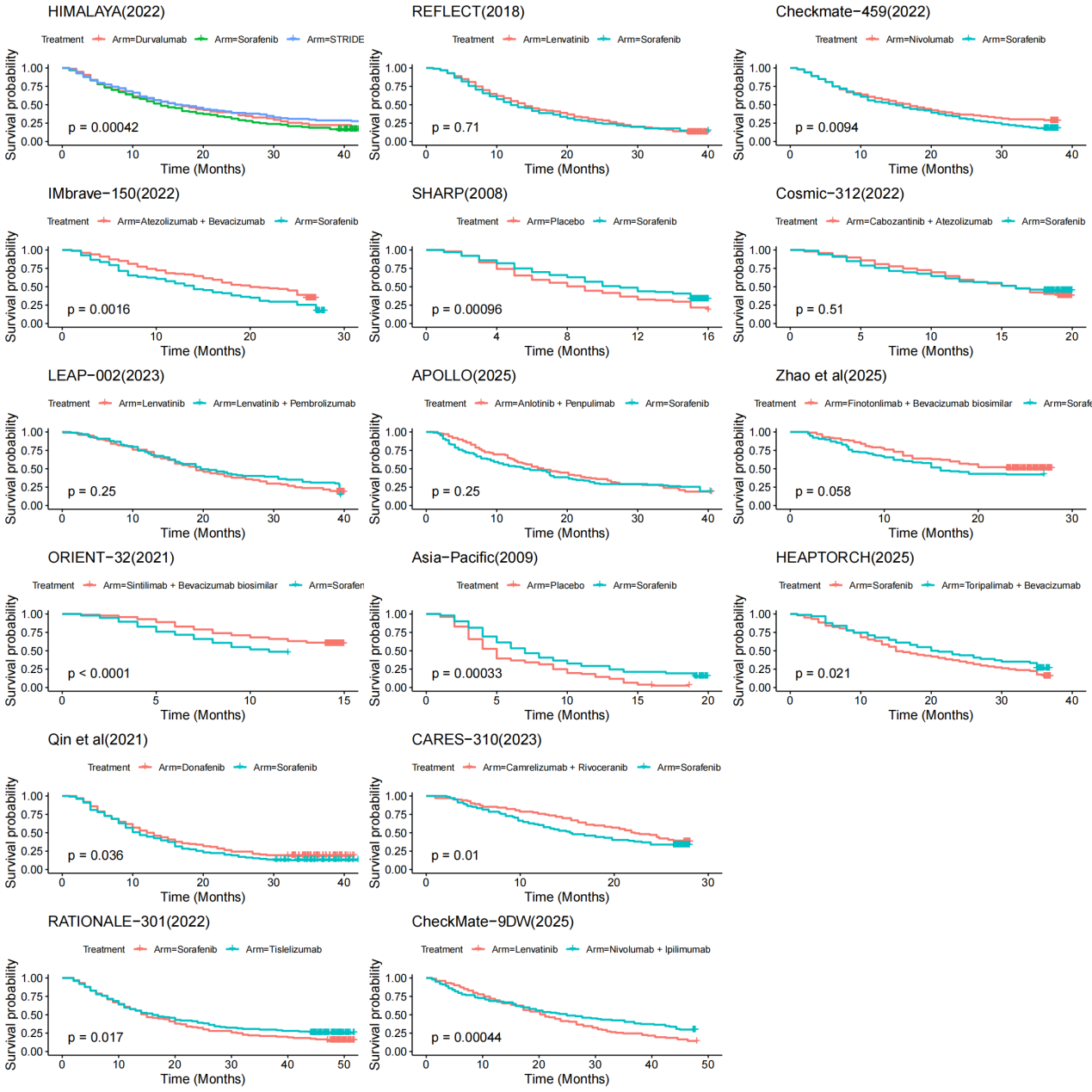


Appendix S4a: Reconstructed IPD for overall survival (OS)


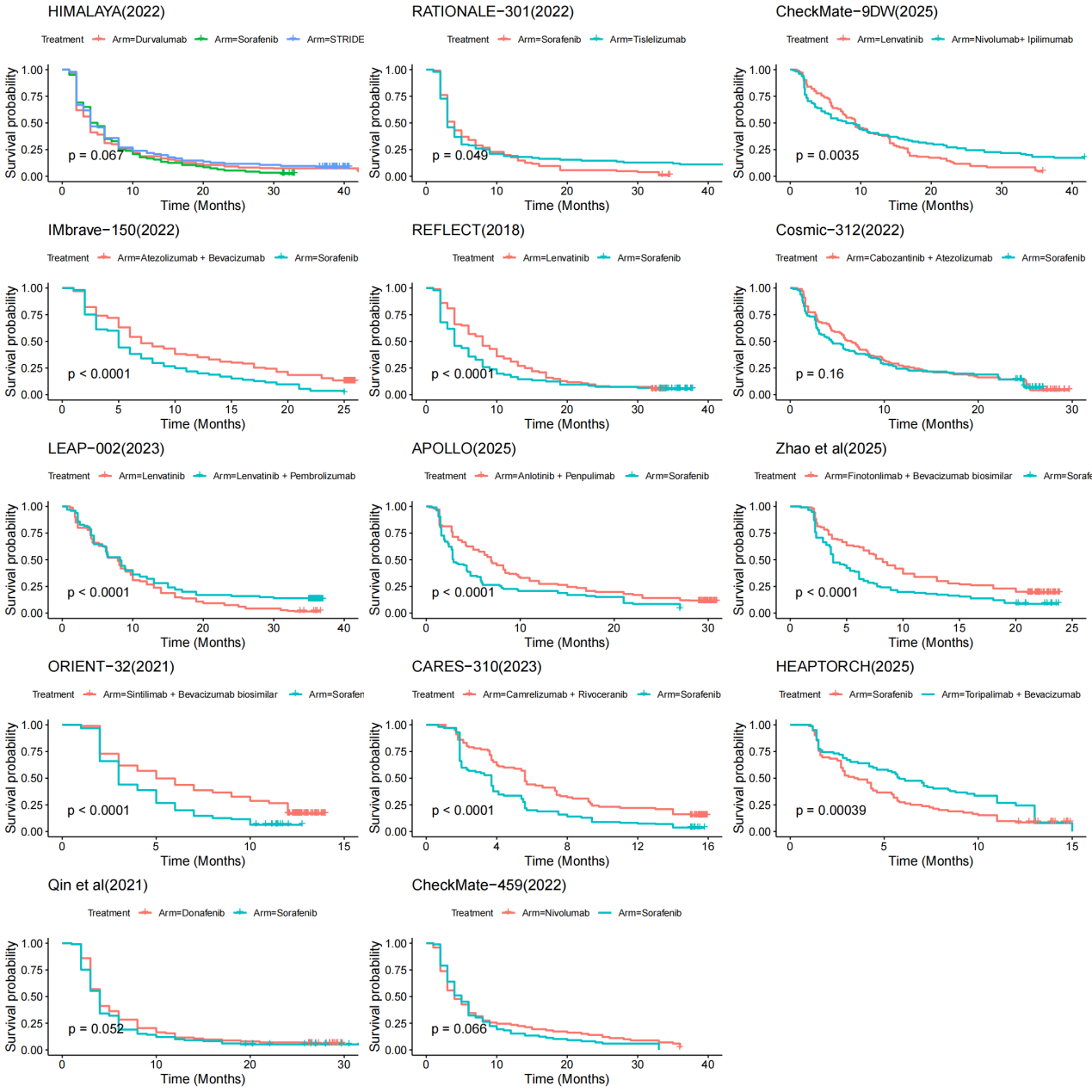


Appendix S4b: Reconstructed IPD for progression-free survival (PFS)

**
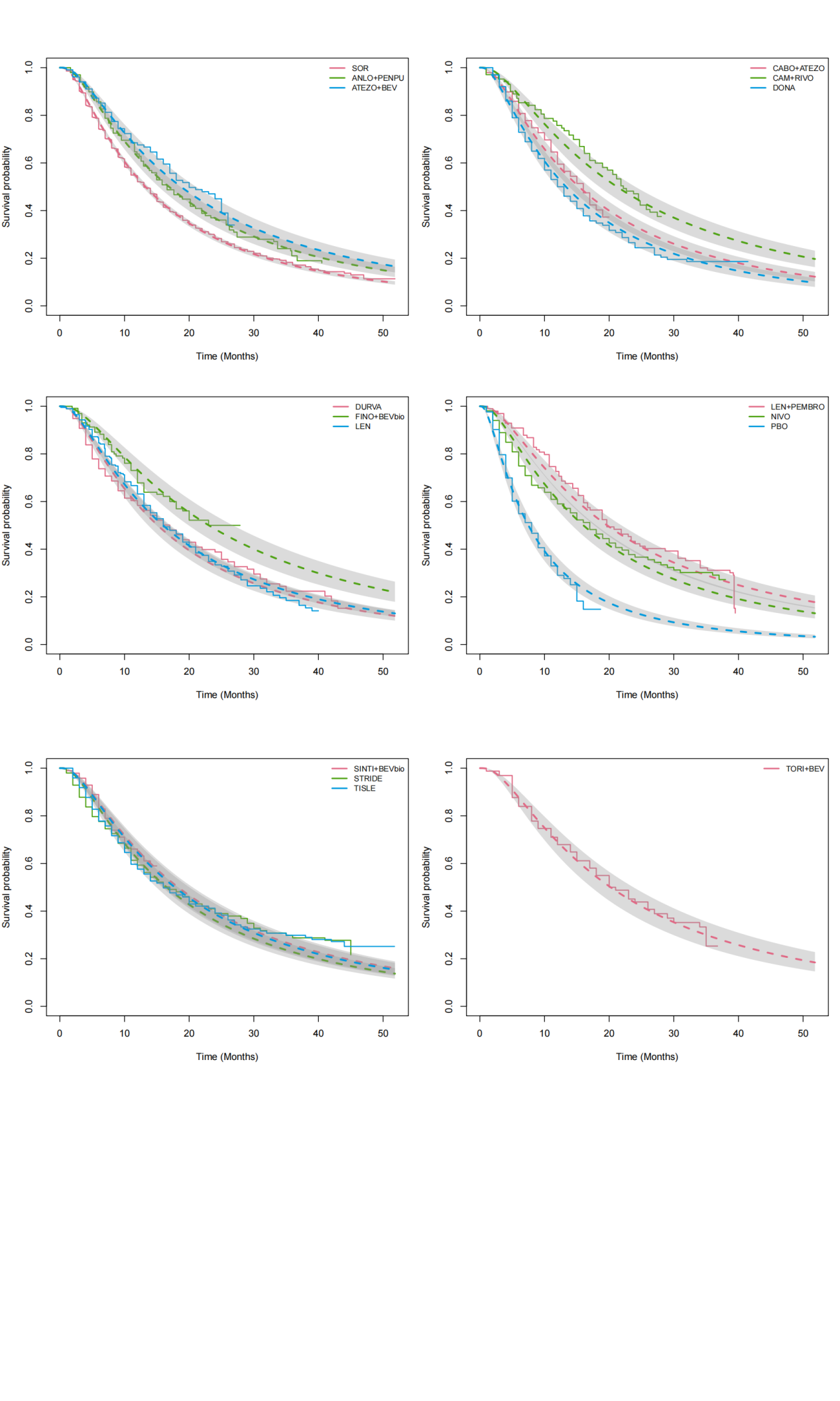
Appendix S5: Observed Kaplan–Meier curves and fitted overall survival (OS) curves estimated using the log-normal parametric survival model**

**Abbreviations:** FINO+BEVbio = finotonlimab + bevacizumab biosimilar; CAM+RIVO = camrelizumab + rivoceranib; TORI+BEV = toripalimab + bevacizumab; LEN+PEMBRO = lenvatinib + pembrolizumab; ATEZO+BEV = atezolizumab + bevacizumab; SINTI+BEVbio = sintilimab + bevacizumab biosimilar; TISLE = tislelizumab; ANLO+PENPU = anlotinib + penpulimab; STRIDE = single tremelimumab regular interval durvalumab regimen; NIVO = nivolumab; LEN = lenvatinib; CABO+ATEZO = cabozantinib + atezolizumab; DURVA = durvalumab; DONA = donafenib; SOR = sorafenib; PBO = placebo;

**
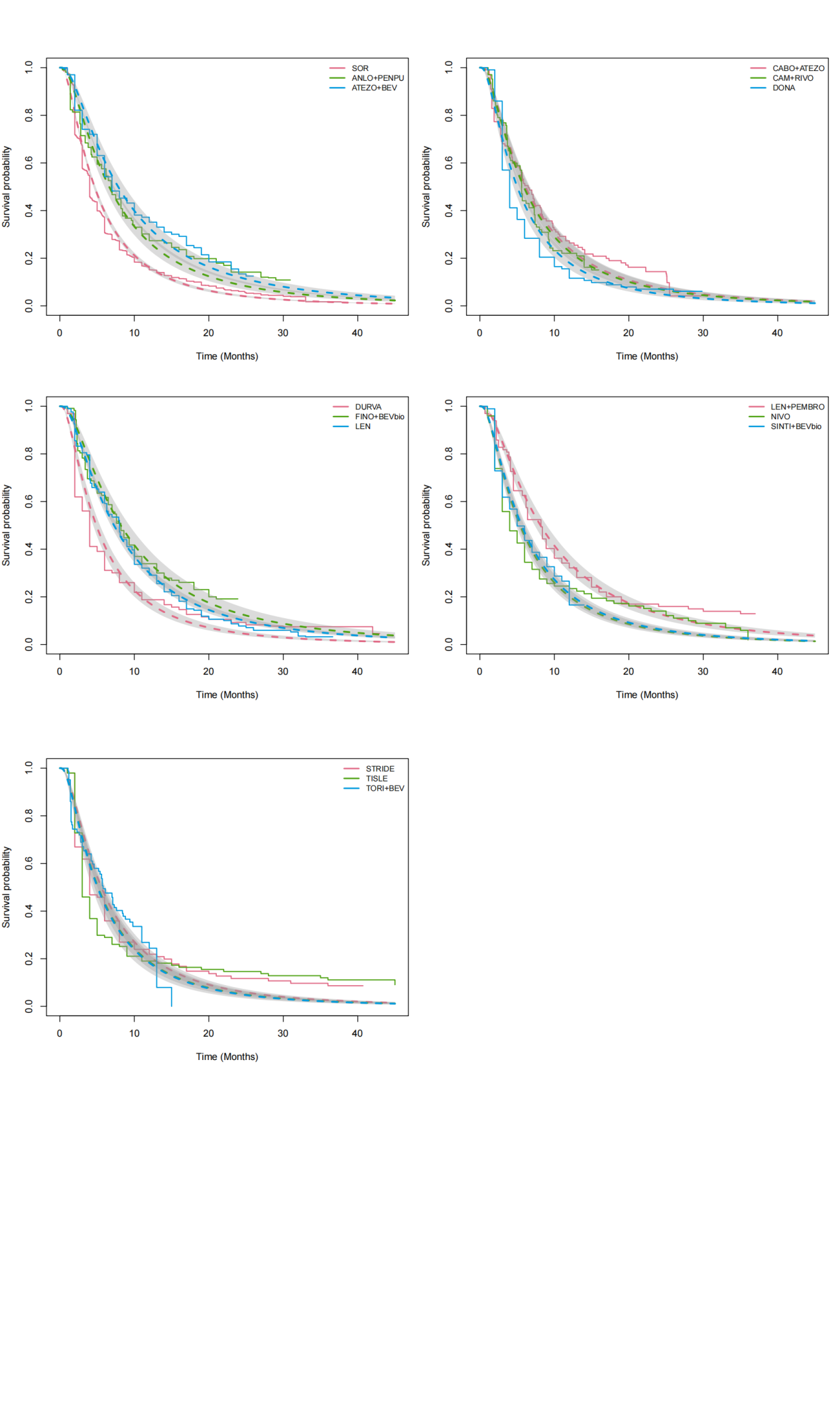
Appendix S6: Observed Kaplan–Meier curves and fitted progression-free survival (PFS) curves estimated using the log-normal parametric survival model**

**Abbreviations:** FINO+BEVbio = finotonlimab + bevacizumab biosimilar; CAM+RIVO = camrelizumab + rivoceranib; TORI+BEV = toripalimab + bevacizumab; LEN+PEMBRO = lenvatinib + pembrolizumab; ATEZO+BEV = atezolizumab + bevacizumab; SINTI+BEVbio = sintilimab + bevacizumab biosimilar; TISLE = tislelizumab; ANLO+PENPU = anlotinib + penpulimab; STRIDE = single tremelimumab regular interval durvalumab regimen; NIVO = nivolumab; LEN = lenvatinib; CABO+ATEZO = cabozantinib + atezolizumab; DURVA = durvalumab; DONA = donafenib; SOR = sorafenib;

**Appendix S7: Fitted survival curves under five parametric distributions**


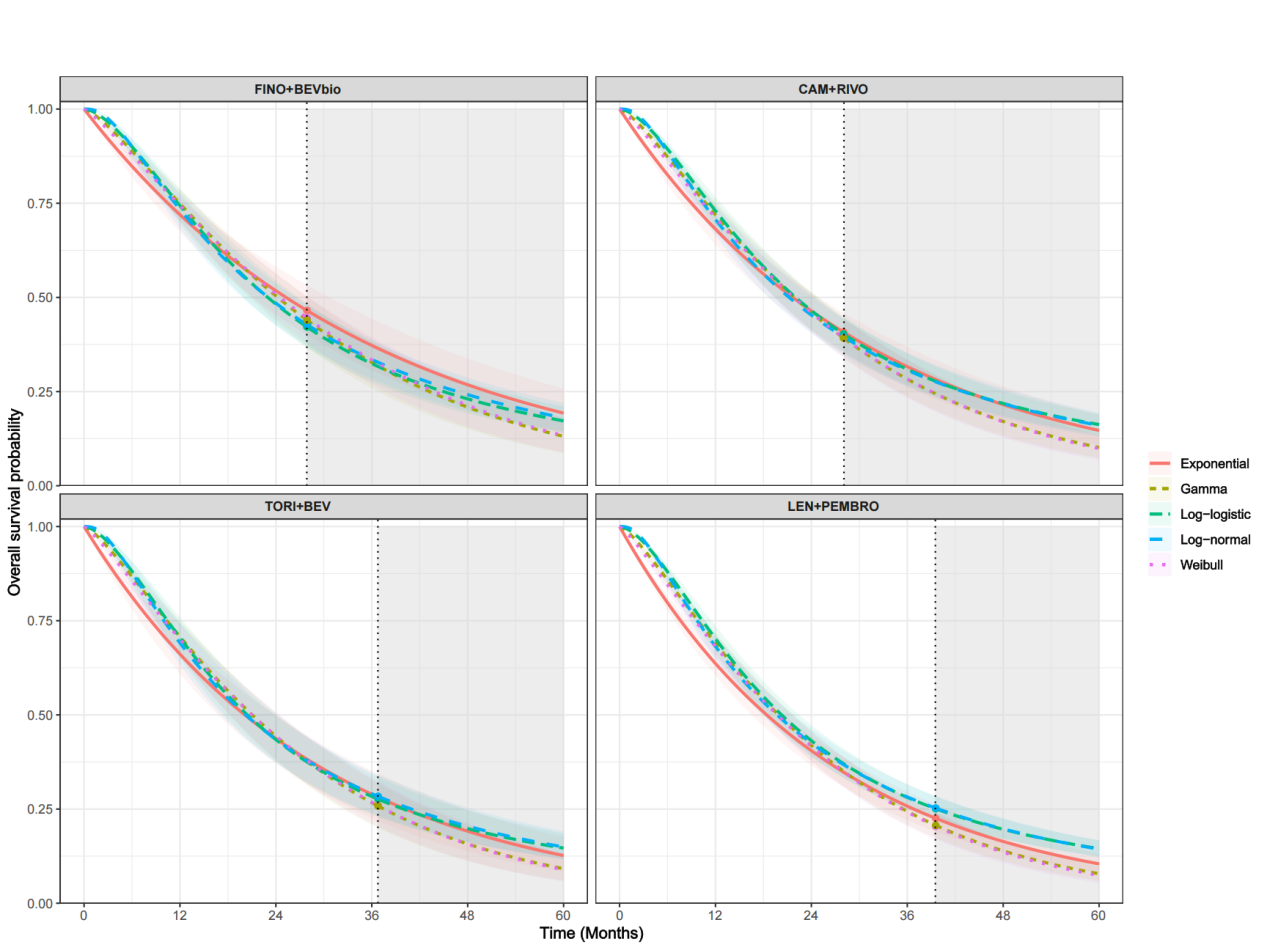


**Abbreviations:** FINO+BEVbio = finotonlimab + bevacizumab biosimilar; CAM+RIVO = camrelizumab + rivoceranib; TORI+BEV = toripalimab + bevacizumab; LEN+PEMBRO = lenvatinib + pembrolizumab;

**
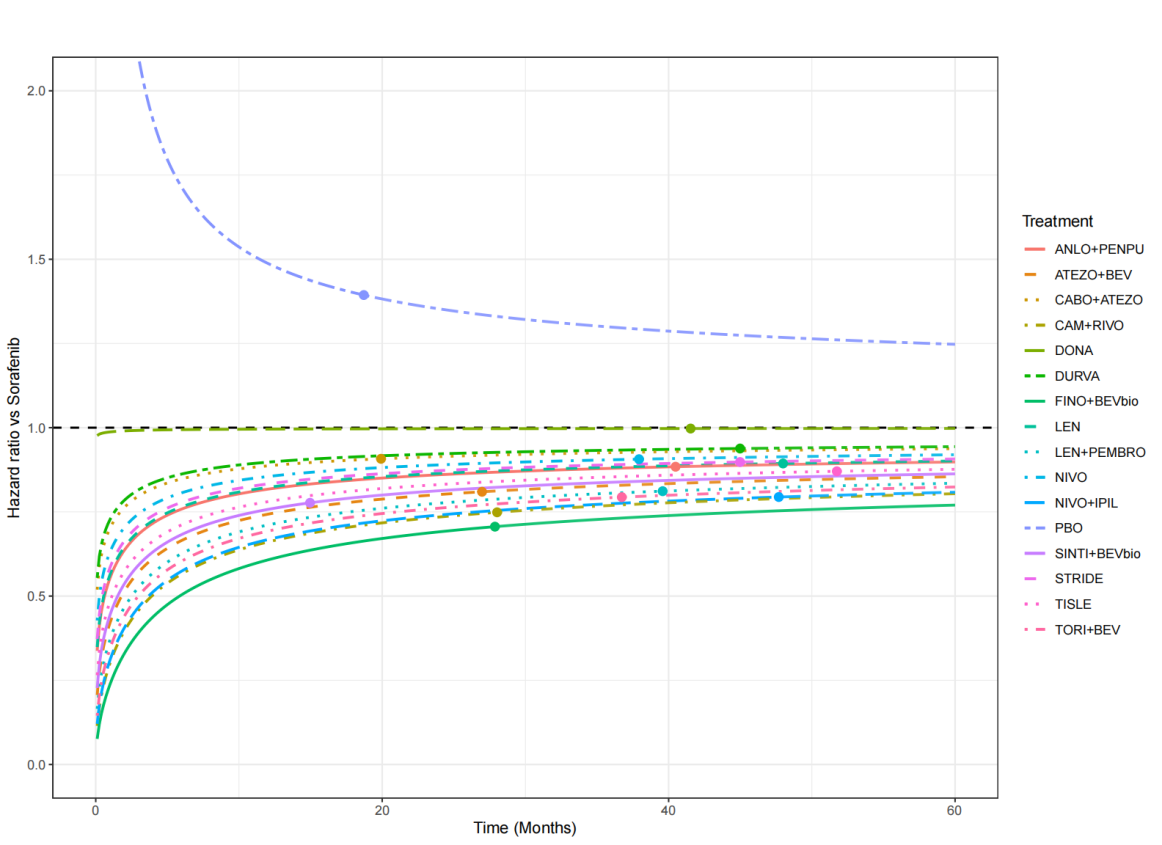
Appendix S8: Sensitivity analysis including CheckMate 9DW: time-varying hazard ratios for overall survival (OS)**

**Abbreviations:** FINO+BEVbio = finotonlimab + bevacizumab biosimilar; CAM+RIVO = camrelizumab + rivoceranib; TORI+BEV = toripalimab + bevacizumab; LEN+PEMBRO = lenvatinib + pembrolizumab; ATEZO+BEV = atezolizumab + bevacizumab; SINTI+BEVbio = sintilimab + bevacizumab biosimilar; TISLE = tislelizumab; ANLO+PENPU = anlotinib + penpulimab; STRIDE = single tremelimumab regular interval durvalumab regimen; NIVO = nivolumab; LEN = lenvatinib; CABO+ATEZO = cabozantinib + atezolizumab; DURVA = durvalumab; DONA = donafenib; SOR = sorafenib; PBO = placebo; NIVO+IPIL = nivolumab + ipilimumab;


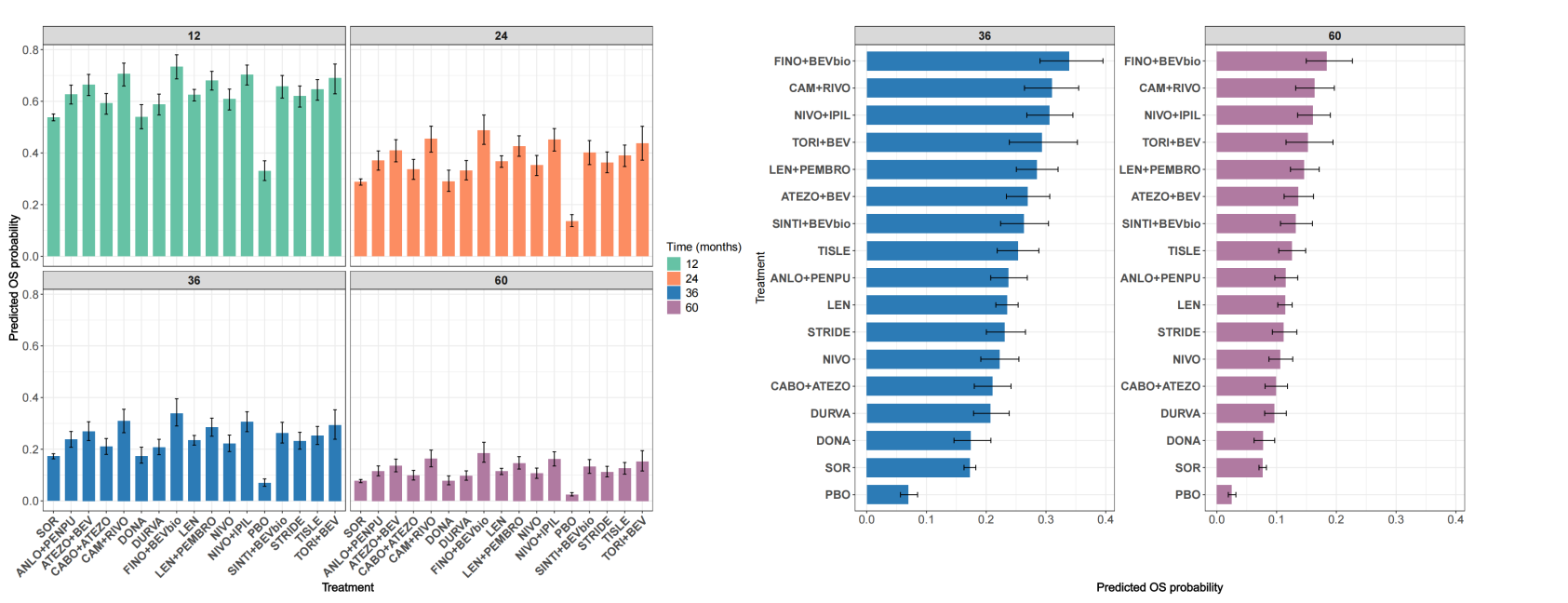
**Appendix S9: Sensitivity analysis including CheckMate 9DW: overall survival (OS) probabilities and ranking**

**Abbreviations:** OS = overall survival; FINO+BEVbio = finotonlimab + bevacizumab biosimilar; CAM+RIVO = camrelizumab + rivoceranib; TORI+BEV = toripalimab + bevacizumab; LEN+PEMBRO = lenvatinib + pembrolizumab; ATEZO+BEV = atezolizumab + bevacizumab; SINTI+BEVbio = sintilimab + bevacizumab biosimilar; TISLE = tislelizumab; ANLO+PENPU = anlotinib + penpulimab; STRIDE = single tremelimumab regular interval durvalumab regimen; NIVO = nivolumab; LEN = lenvatinib; CABO+ATEZO = cabozantinib + atezolizumab; DURVA = durvalumab; DONA = donafenib; SOR = sorafenib; PBO = placebo; NIVO+IPIL = nivolumab + ipilimumab;

**
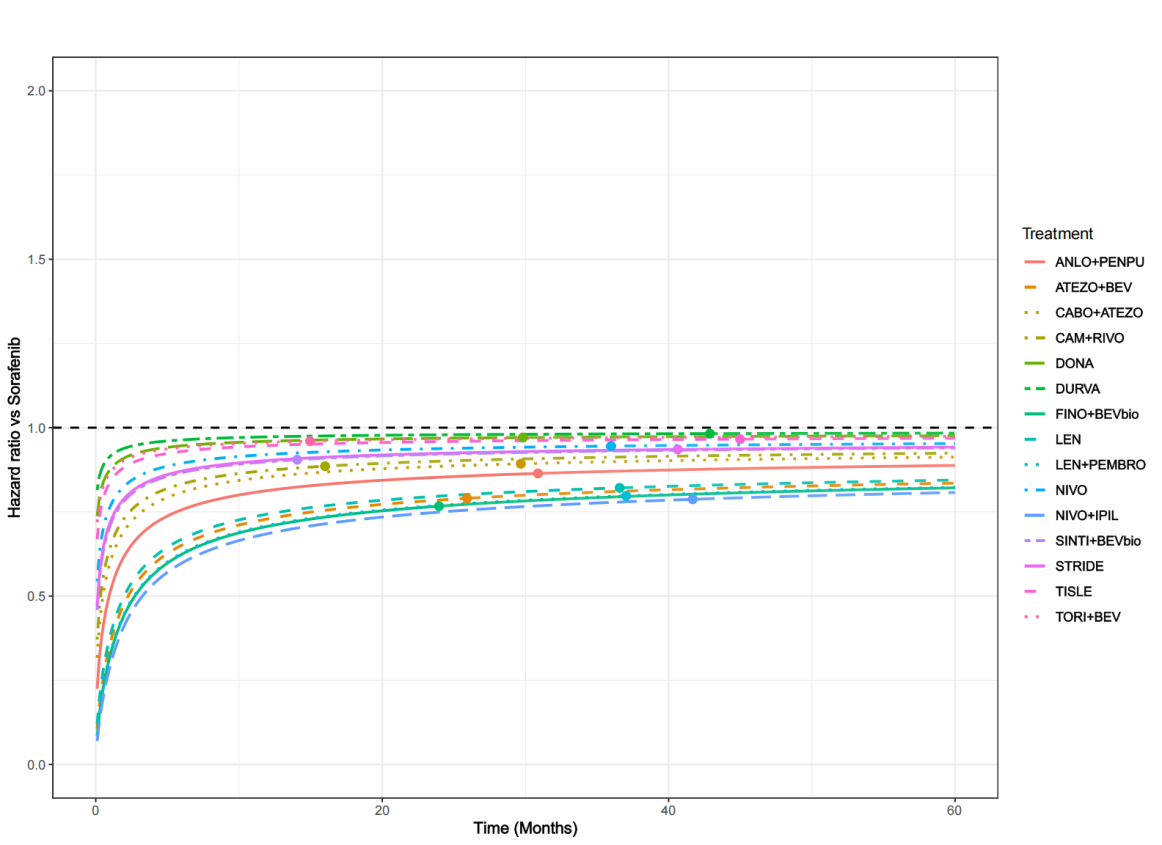
Appendix S10: Sensitivity analysis including CheckMate 9DW: time-varying hazard ratios for progression-free survival (PFS)**

**Abbreviations:** ANLO+PENPU = anlotinib + penpulimab; ATEZO+BEV = atezolizumab + bevacizumab; CABO+ATEZO = cabozantinib + atezolizumab; CAM+RIVO = camrelizumab + rivoceranib; DONA = donafenib; DURVA = durvalumab; FINO+BEVbio = finotonlimab + bevacizumab biosimilar; LEN = lenvatinib; LEN+PEMBRO = lenvatinib + pembrolizumab; NIVO = nivolumab; SINTI+BEVbio = sintilimab + bevacizumab biosimilar; STRIDE = single tremelimumab regular interval durvalumab regimen; TISLE = tislelizumab; TORI+BEV = toripalimab + bevacizumab; NIVO+IPIL = nivolumab + ipilimumab;

**Appendix S11: Sensitivity analysis including CheckMate 9DW: progression-free survival (PFS) probabilities and ranking**

**
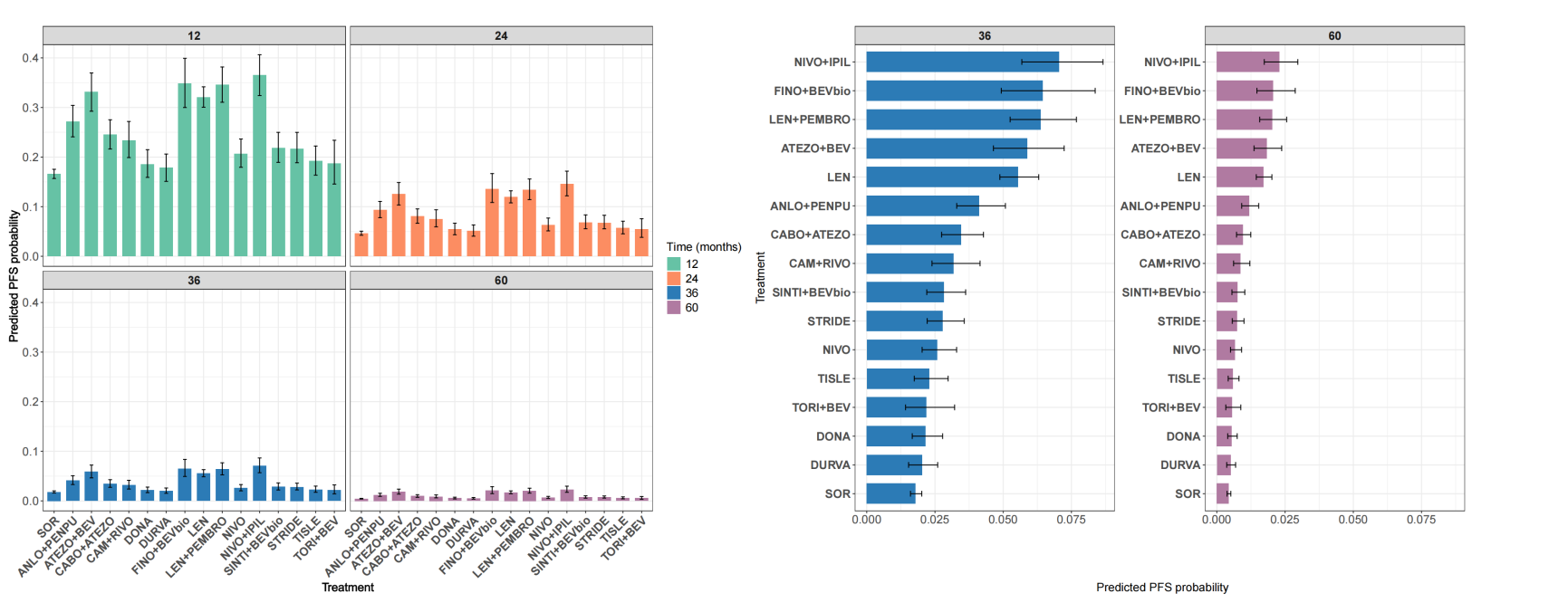
Abbreviations:** PFS = progression-free survival; ANLO+PENPU = anlotinib + penpulimab; ATEZO+BEV = atezolizumab + bevacizumab; CABO+ATEZO = cabozantinib + atezolizumab; CAM+RIVO = camrelizumab + rivoceranib; DONA = donafenib; DURVA = durvalumab; FINO+BEVbio = finotonlimab + bevacizumab biosimilar; LEN = lenvatinib; LEN+PEMBRO = lenvatinib + pembrolizumab; NIVO = nivolumab; SINTI+BEVbio = sintilimab + bevacizumab biosimilar; STRIDE = single tremelimumab regular interval durvalumab regimen; TISLE = tislelizumab; TORI+BEV = toripalimab + bevacizumab; NIVO+IPIL = nivolumab + ipilimumab;

**Appendix S12: Summary of safety outcomes and major adverse events in patients with uHCC**

| Study | Treatment arm | Any event | Grade ≥3 TRAEs | SAE | Discontinuation due to AE | Treatment-related deaths | Major toxicities |
| --- | --- | --- | --- | --- | --- | --- | --- |
| Llovet et al (2008)  SHARP | Sorafenib (n=297) | 80%  (n=238) | 20%  (n=60) | 52%  (n=153) | 11%  (n=34) | NR | Diarrhea, fatigue, HFSR, weight loss, rash |
|  | Placebo (n=302) | 52%  (n=157) | <5%  (n<15) | 54%  (n=164) | 5%  (n=15) | NR | Diarrhea, fatigue, rash |
| Cheng et al (2009)  Asia-Pacific | Sorafenib (149 analysed for safety) | 81.9%  (n=122) | NR | 8.7%  (n=13) | 19.5%  (n=29) | NR | HFSR, diarrhea, alopecia, fatigue, anorexia |
|  | Placebo (75 analysed for safety) | 38.7%  (n=29) | NR | 1.3%  (n=1) | 13.3%  (n=10) | NR | Lower overall incidence than sorafenib |
| Kudo et al (2018)  REFLECT | Lenvatinib (n=476) | 99% (n=470) | 57%  (n=270) | 18%  (n=84) | 9%  (n=42) | 2%  (n=11) | Hypertension, weight decrease, proteinuria, appetite decrease, diarrhea |
|  | Sorafenib (n=475) | 99% (n=472) | 49%  (n=231) | 10%  (n=48) | 7%  (n=34) | 1%  (n=4) | HFSR, AST increase, bilirubin increase, hypertension |
| Cheng AL et al  (2022)  IMbrave | Atezolizumab + Bevacizumab (n=329) | 98%  (n=322) | 56%  (n=184) | 38%  (n=125) | 15% (n=49) | 2%  (n=6) | Hypertension, proteinuria, AST increase,  fatigue, haemorrhage |
|  | Sorafenib (n=156) | 99%  (n=154) | 55%  (n=86) | 30%  (n=47) | 10%  (n=16) | 1%  (n=2) | HFSR, diarrhea, fatigue, AST/ALT increase |
| Yau et al (2022)  CheckMate-459 | Nivolumab (367 analysed for safety) | 81.2% (n=298) | 22% (n=81) | 12%  (n=43) | 4%  (n=15) | 1%  (n=4) | AST increase , hepatitis,fatigue, pruritus, rash |
|  | Sorafenib (363 analysed for safety) | 92.0% (n=334) | 49%  (n=178) | 11%  (n=39) | 8%  (n=29) | <1%  (n=1) | HFSR, hypertension, AST increase , diarrhea, fatigue |
| Abou-Alfa et al (2022)  HIMALAYA | Tremelimumab + Durvalumab (STRIDE; n=388) | 97.4% (n=378) | 50.5% (n=196) | 40.5% (n=157) | 13.7%  (n=53) | 7.7%  (n=30) | Hepatic events, diarrhea/colitis, dermatitis/rash; immune-mediated AEs (high-dose steroids) |
|  | Durvalumab (n=388) | 88.9% (n=345) | 37.1%  (n=144) | 29.6%  (n=115) | 8.2%  (n=32) | 6.7%  (n=26) | Diarrhea, rash, pruritus, fatigue, hypothyroidism, AST/ALT increase |
|  | Sorafenib (n=374) | 95.5% (n=357) | 52.4%  (n=196) | 29.7%  (n=111) | 16.8%  (n=63) | 7.2%  (n=27) | Palmar-plantar erythrodysesthesia syndrome; Diarrhea; Hypertension |
| Ren et al (2021)  ORIENT-32 | Sintilimab + Bevacizumab biosimilar (IBI305) (n=380) | 98.9% (n=376) | NR | 32%  (n=123) | 14%  (n=52) | 3%  (n=10) | Hypertension, proteinuria, AST/ALT increase, bilirubin, hypothyroidism |
|  | Sorafenib (n=185) | 97.8% (n=181) | NR | 19%  (n=36) | 6%  (n=11) | 3%  (n=6) | HFSR, diarrhea, fatigue |
| Qin et al (2023)  CARES-310 | Camrelizumab + Rivoceranib (n=272) | 99.6% (n=271) | 81%  (n=220) | 24%  (n=66) | 24%  (n=66) | <1%  (n=1) | Hypertension, AST increase, ALT increase, proteinuria, RCCEP |
|  | Sorafenib (n=269) | 98.5% (n=265) | 52% (n=141) | 6%  (n=16) | 4%  (n=12) | <1%  (n=1) | Hypertension, HFSR, diarrhea, AST increase |
| Yau et al (2025)  CheckMate-9DW | Nivolumab + Ipilimumab (n=335) | 83.7% (n=278) | 41%  (n=137) | NR | 18%  (n=59) | 4%  (n=12) | Hepatitis, rash, hypothyroidism, fatigue, diarrhea, adrenal insufficiency (immune-mediated) |
|  | Lenvatinib or Sorafenib (n=325) | 91.4% (n=297) | 42%  (n=138) | NR | 10%  (n=34) | 1%  (n=3) | HFSR, diarrhea, fatigue, hypertension, AST/ALT increase |
| Kelley et al (2022)  COSMIC-312 | Cabozantinib + Atezolizumab (n=429) | 99.8% (n=428) | 76%  (n=285) | 18%  (n=78) | 14%  (n=58) | NR | HFSR, hypertension, diarrhea, fatigue, ALT increase, nausea |
|  | Sorafenib (n=207) | 99.0% (n=205) | 57%  (n=118) | 8%  (n=16) | 8%  (n=16) | NR | HFSR, diarrhea, fatigue, hypertension |
| Llovet JM et al (2023)  LEAP-002 | Lenvatinib + Pembrolizumab (n=395) | 96.5% (n=381) | 63%  (n=247) | 25%  (n=99) | 18%  (n=71) | 1%  (n=4) | Hypothyroidism, hypertension, diarrhea, HFSR, appetite decrease , AST increase |
|  | Lenvatinib + Placebo (n=395) | 95.7% (n=378) | 58%  (n=227) | 16%  (n=65) | 11%  (n=42) | 1%  (n=3) | Hypothyroidism, HFSR, hypertension , diarrhea |
| Qin et al (2021) | Donafenib (n=333) | 99.7% (n=332) | 38% (n=125) | 7%  (n=23) | 10%  (n=34) | 2%  (n=6) | diarrhea, HFSR, fatigue, liver dysfunction |
|  | Sorafenib (n=332) | 99.1% (n=329) | 50%  (n=165) | 7%  (n=22) | 13%  (n=42) | 4%  (n=12) | HFSR, diarrhea, alopecia, hypertension, fatigue |
| Qin et al (2022)  RATIONALE-301 | Tislelizumab (n=342) | 95% (n=325) | 48.2%  (n=163) | 29.9%  (n=101) | 10.9%  (n=37) | 4.4%  (n=15) | Hepatitis,hypothyroidism, AST/ALT increase, fatigue, pruritus |
|  | Sorafenib (n=332) | 97.6% (n=324) | 65.4%  (n=212) | 28.1% (n=91) | 18.5%  (n=60) | 5.2%  (n=17) | HFSR, hypertension, diarrhea |
| Shi et al (2025)  HEPATORCH | Toripalimab + Bevacizumab (n=162) | 98% (n=158) | 63%  (n=102) | 41%  (n=66) | 13%  (n=21) | 1.2%  (n=2) | Upper GI haemorrhage, abnormal hepatic function, ascites, hypothyroidism, hyponatraemia |
|  | Sorafenib (n=164) | 99% (n=162) | 61%  (n=100) | 29%  (n=47) | 12%  (n=20) | 0.6%  (n=1) | HFSR, diarrhea, fatigue, AST increase |
| Zhou et al (2025)  APOLLO | Anlotinib + Penpulimab (n=432) | 99.1% (n=428) | 59% (n=257) | 21%  (n=90) | 9%  (n=40) | <1%  (n=1) | Hypertension, platelet decrease, AST increase, proteinuria, hypothyroidism |
|  | Sorafenib (n=211) | 99.1% (n=209) | 55% (n=116) | 9%  (n=19) | 4%  (n=8) | 1%  (n=2) | Hypertension, HFSR, diarrhea, platelet decrease, AST increase |
| Zhao et al (2025) | Finotonlimab + Bevacizumab biosimilar (n=230) | 97.0% (n=223) | 52.6%  (n=121) | 29.1%  (n=67) | 13%  (n=30) | 3%  (n=7) | Proteinuria, platelet decrease, hypertension, ALT/AST increase, fatigue |
|  | Sorafenib (n=116) | 93.1% (n=108) | 37.9% (n=44) | 12.9%  (n=15) | 4.3%  (n=5) | 3%  (n=2) | Proteinuria, HFSR, diarrhea, fatigue |

***Note:*** *Safety outcome definitions were based on the original trial reports and were not fully uniform across studies. Major toxicities were extracted as reported in each individual trial without reclassification, to preserve the original safety reporting. Values in the “Any event” column were extracted directly from the original publications. Due to differences in reporting standards across trials, direct comparisons of specific toxicities were not performed.*

**Abbreviations:** AE = adverse event; ALT = alanine aminotransferase; AST = aspartate aminotransferase; GI = gastrointestinal; HFSR = hand-foot skin reaction; NR = not reported; RCCEP = reactive cutaneous capillary endothelial proliferation; SAE = serious adverse event; TRAE = treatment-related adverse event; uHCC = unresectable hepatocellular carcinoma.
